# Elimination of huntingtin in the adult mouse leads to progressive behavioral deficits, bilateral thalamic calcification, and altered brain iron homeostasis

**DOI:** 10.1371/journal.pgen.1006846

**Published:** 2017-07-17

**Authors:** Paula Dietrich, Irudayam Maria Johnson, Shanta Alli, Ioannis Dragatsis

**Affiliations:** Department of Physiology, The University of Tennessee, Health Science Center, Memphis, Tennessee, United States of America; CHDI Foundation, UNITED STATES

## Abstract

Huntington’s Disease (HD) is an autosomal dominant progressive neurodegenerative disorder characterized by cognitive, behavioral and motor dysfunctions. HD is caused by a CAG repeat expansion in exon 1 of the HD gene that is translated into an expanded polyglutamine tract in the encoded protein, huntingtin (HTT). While the most significant neuropathology of HD occurs in the striatum, other brain regions are also affected and play an important role in HD pathology. To date there is no cure for HD, and recently strategies aiming at silencing HTT expression have been initiated as possible therapeutics for HD. However, the essential functions of HTT in the adult brain are currently unknown and hence the consequence of sustained suppression of HTT expression is unpredictable and can potentially be deleterious. Using the Cre-loxP system of recombination, we conditionally inactivated the mouse HD gene homologue at 3, 6 and 9 months of age. Here we show that elimination of Htt expression in the adult mouse results in behavioral deficits, progressive neuropathological changes including bilateral thalamic calcification, and altered brain iron homeostasis.

## Author summary

Huntington’s Disease is a genetic disorder characterized by progressive cognitive, behavioral and motor dysfunctions. Usually the first symptoms appear around 40 years of age, and lead to death within 15–20 years after the onset of symptoms. To date there is no cure for Huntington’s Disease, and current therapeutic strategies are only palliative, and far from optimal. Gene silencing currently appears as the most attractive approach for the treatment of Huntington’s Disease. However, since normal and mutant huntingtin (the protein product of the Huntington’s disease gene) differ only on the polyglutamine length, unless allele-specific silencing is planned, normal huntingtin (that is neuroprotective) will also be inactivated with unknown implications. To address these questions, we investigated the consequences of elimination of normal huntingtin function in adulthood. In summary, our studies show that huntingtin plays a role in brain iron homeostasis, and that elimination of huntingtin in the adult mouse results in behavioral deficits and progressive neuropathological changes including bilateral thalamic calcification.

## Introduction

Huntington’s Disease (HD, OMIM#143100) is an autosomal dominant progressive neurodegenerative disorder characterized by cognitive, behavioral and motor dysfunctions. The mean age of onset of HD is around 40 years, and progression of the disorder usually leads to death within 15–20 years after the onset of symptoms [1, 2].

HD is caused by a trinucleotide (CAG) repeat expansion in exon 1 of the HTT gene that is translated into an expanded polyglutamine tract in the amino-terminal region of the encoded protein, huntingtin (HTT). The toxic effects of mutant HTT protein depend on the size of the polyglutamine tract, and there is an inverse correlation between age-of-onset of HD symptoms and CAG repeat size, with more than 35 CAG repeats leading to neuronal intranuclear inclusions, neuronal cellular dysfunction and cell death [2, 3].

The earliest and most significant neuropathology of HD is observed in the striatum [2, 4]. However, other brain regions are also affected and play an important role in HD pathology. Significant functional alterations in neurotransmitter release, neuronal cell loss, gliosis and shrinkage also occur in the cerebral cortex, hypothalamus, thalamus, and substantia nigra, and by end-stage HD patients have lost more than 30% of their total brain mass [4, 5].

To date there is no cure for HD, and current therapeutic strategies are only palliative, and far from optimal [6]. Gene silencing currently appears as the most attractive approach for the treatment of HD [6–8]. The vast majority of the HD patients are heterozygous for the CAG expansion allele. However, since normal and mutant HTT differ only in the CAG repeat length, unless allele-specific silencing is planned, normal HTT levels will also be reduced with unknown implications. Recent studies in animal models of HD have shown that HD phenotypes are ameliorated or reversed when expression of mutant Htt is downregulated in the striatum, even if endogenous normal Htt levels are simultaneously reduced [9–12]. In fact and on the premises of the above-mentioned studies, clinical trials using antisense technology that will silence both the mutant and normal HTT are already underway [6, 8, NCT02519036]. However, in these recent animals studies [9–12], the effects of sustained Htt silencing in the brain was not evaluated. Answers to these questions are highly relevant to therapeutics since normal Htt is neuroprotective [13–15] and Htt elimination in the developing or early postnatal mouse brain leads to neurodegeneration and impaired adult neuronal morphology [16,17].

To address these questions, we investigated the consequences of elimination of normal Htt function in adulthood. Using the Cre-loxP system of recombination, we conditionally inactivated the mouse HTT gene homologue (Hdh) at 3, 6 and 9 months of age. Our results indicate that elimination of Htt expression in the adult mouse leads to severe progressive behavioral and motor impairments, and widespread neuropathology including progressive bilateral thalamic calcification. Furthermore, our results also suggest that altered brain iron homeostasis is a contributing factor for the neuropathology and behavioral deficits associated with Htt elimination.

## Results

### Conditional inactivation of the Hdh gene in the adult mouse

**Fig 1:**
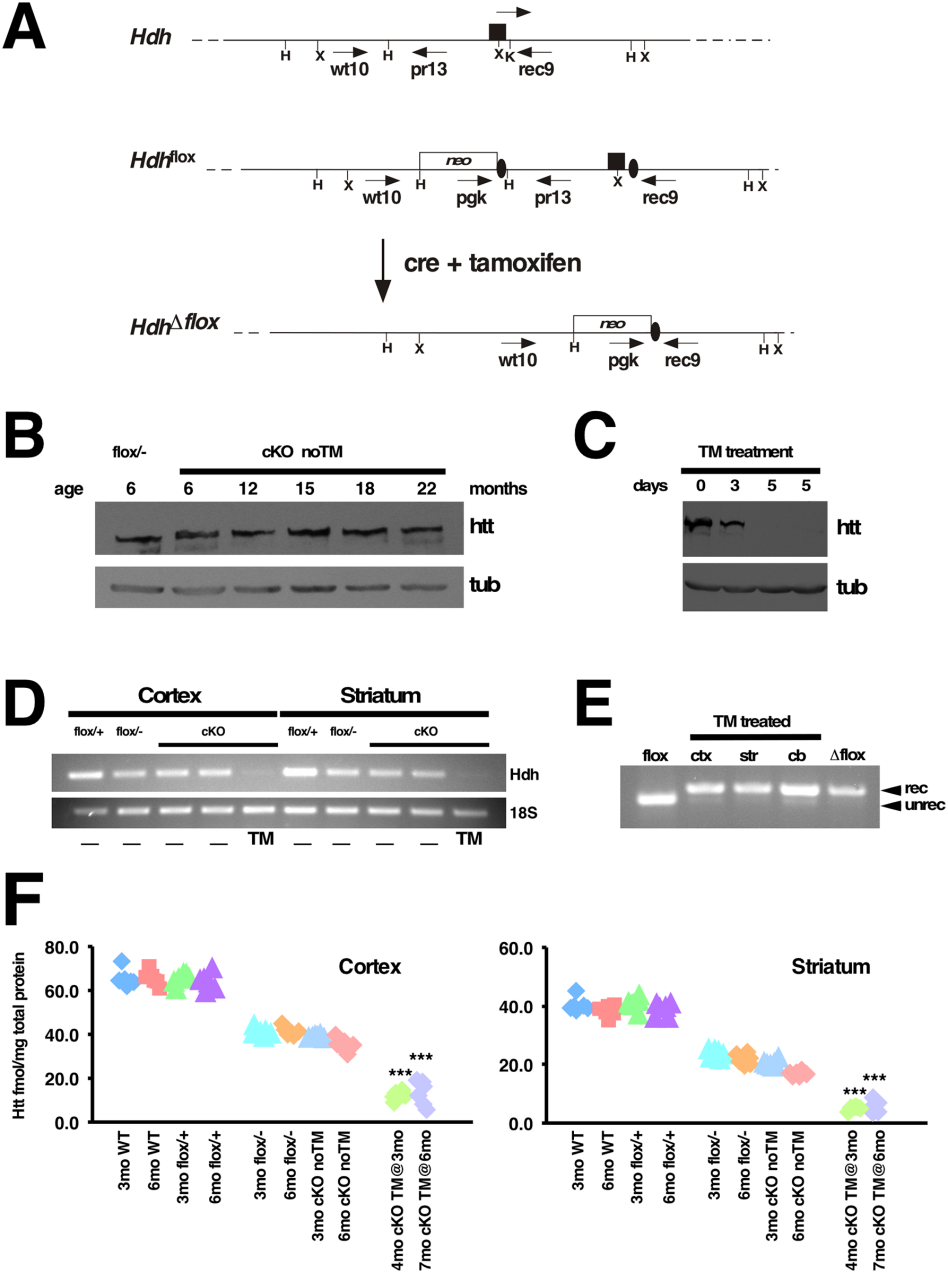
Generation and evaluation of a tamoxifen inducible system for huntingtin ablation in the adult mouse. (A) Schematic representation of wild-type allele (Hdh), Hdh loxP flanked allele (Hdh^flox^), and Hdh allele after tamoxifen-induced cre-mediated recombination (Hdh^Δflox^). Exon 1 is represented by a black rectangle, and black ovals represent the loxP sites. Restriction sites shown on the schematic are: HindIII (H), XmnI (X) and KpnI (K). The location of oligonucleotides used for genotyping is indicated under arrows. (B) Western blot of total protein extracts from brains of 6 month-old *Hdh*^*flox/-*^ (flox/-), and untreated CreER; *Hdh*^*flox/-*^ (cKO noTM) mice at 6, 12, 15, 18, and 22 months of age was probed with mouse monoclonal anti-htt 2166 antibody (Chemicon), stripped and re-probed with anti-β-tubulin antibody (tub, lower panel) to ensure equal loading of the samples. Note that Htt expression in untreated cKO mice does not decrease significantly over time, and also does not differ from that of *Hdh*^*flox/-*^ (flox/-) controls. (C) Western blot of total brain protein extracts from cKO noTM mouse (lane 1), cKO mouse TM-treated for 3 consecutive days (lane 2), and cKO mice TM-treated for 5 consecutive days (lanes 3 and 4) probed with mouse monoclonal anti-htt 2166 antibody (Chemicon) (Htt, top panel), stripped and re-probed with anti-β-tubulin antibody (tub, lower panel) to ensure equal loading of the samples. Note that the Htt band is significantly reduced after 3 days of TM (lane 2) and barely detectable after 5 days of TM administration (lanes 3 and 4). (D) Assessment of Hdh mRNA expression level by RT-PCR. Total RNA from cortex and striatum from *Hdh*^*flox/+*^ (flox/+); *Hdh*^*flox/-*^ (flox/-); non-treated and TM-treated cKO mice was extracted and reverse transcribed. Semi-quantitative PCR amplification was performed using primers specific for Hdh coding regions spanning exons 11 and 12 (top panel) and 18S rRNA for internal control (bottom panel). (E) Total genomic DNA from cortex (ctx), striatum (str), and cerebellum (cb) from TM treated cKO mouse was submitted to PCR using primers that amplify the unrecombined (unrec) flox allele and recombined (rec) Δflox Hdh allele. Genomic DNA from *Hdh*^*flox/+*^ brain (flox: lane 1) and fully recombined Δflox (lane 5) were also submitted to PCR amplification with the same primers in parallel, as controls. Note that the unrecombined PCR product is barely visible in samples after TM treatment. (F) Quantification of htt protein levels using MSD electrochemiluminescence. Htt protein levels were independently evaluated and determined by the biopharmaceutical company BioFocus (A Galapagos Company). Cortices (left graph) and striata (right graph) from 3 month-old and 6 month-old C57BL/6 WT (3mo WT, 6mo WT accordingly), *Hdh*^*flox/+*^ (flox/+), *Hdh*^*flox/-*^ (flox/-), untreated cKO (cKO noTM) and cKO TM-treated at 3 months of age and harvested at 4 months (4mo cKO TM@3mo) or cKO TM-treated at 6 months of age and harvested at 7 months (7mo cKO TM @6mo) were analyzed. For each time-point, genotype and treatment, 3 female and 3 male mice were analyzed. Individual data are depicted in these scatter plots and htt is expressed in fmol/mg of total protein. Note that the group 4mo cKO TM@3mo has only 5 samples, since one of the samples was wrongly genotyped and was therefore not included in the plots. Tamoxifen-induced cre-mediated recombination results in severe reduction of htt levels (***P<0.001, Student’s t-test).

To investigate the need of Htt in the adult mouse, we conditionally inactivated the Hdh gene globally, using the tamoxifen-inducible Cre transgenic line CAGG-CreER^™^ [18]. This transgenic line ubiquitously expresses the Cre recombinase fused with a mutated ligand binding-domain of the estrogen receptor (ER), giving rise to a tightly regulated protein whose activity is induced by tamoxifen. For our breeding strategy, *Hdh*^*+/-*^ mice [19] were crossed with CAGG-CreER^™^ (hereafter referred as CreER) mice and the resulting CreER; *Hdh*^*+/-*^ progeny was then crossed with *Hdh*^*flox/flox*^ mice [16] to generate CreER; *Hdh*^*flox/-*^ mice and controls. As described in Dragatsis et al., 2000 (Fig 1A) the Hdh flox allele contains one loxP site inserted within intron 1 and a second loxP site located 1.3kb upstream of the Hdh transcription initiation site, so that upon Cre-mediated recombination the promoter region and exon 1 are deleted and a null allele is generated. CreER; *Hdh*^*flox/-*^ (hereafter referred as cKO) mice were born in Mendelian ratio and were indistinguishable from control littermates from birth until adulthood, suggesting that little if any leaky Cre-mediated recombination occurs in the absence of tamoxifen induction.

Western analyses confirmed that Htt expression in cKO brains at 6, 12, and 15 months of age was comparable to that of control *Hdh*^*flox/-*^ mice (Fig 1B and S1A Fig), indicating therefore that the system is tight enough to analyze the effects of Htt ablation in the adult. Efficient tamoxifen (TM)-induced recombination and consequent elimination of Htt expression in cKO mice was achieved by intraperitoneal injection of 2.0–3.0 mg TM/26 g of body weight for five consecutive days. TM-induced elimination of Htt expression was verified by western blot analyses on total protein extracts derived from forebrain of untreated and TM-treated cKO mice (Fig 1C and S1B Fig), as well as by semi-quantitative RT-PCR (Fig 1D and S1C Fig). On average, intraperitoneal injections of tamoxifen for five consecutive days in cKO mice resulted in almost complete elimination of Htt expression in the brain as assessed by western analyses (Fig 1C). Similar results were obtained by MSD electrochemiluminescence assays performed independently by the biopharmaceutical company BioFocus (Fig 1F). The residual Htt expression observed in cortex and striatum is likely due either to lack of Htt ablation in specific cell types or to a small level of mosaicism across the different cell populations.

As expected, similar to *Hdh*^*flox/-*^ mice, untreated cKO mice express about 50% the levels of Htt compared to WT or *Hdh*^*flox/+*^ mice (Fig 1F and S1C Fig). Consistent with the nearly complete elimination of Htt expression observed at the protein and RNA level, extensive recombination of the Hdh flox allele was observed at the DNA level in cortex, striatum as well as in the cerebellum of TM-treated CreER; *Hdh*^*flox/-*^ mice (Fig 1E). Cre-mediated recombination upon tamoxifen injection also occurred with high efficiency in the spinal cord and peripheral organs (S1D Fig).

### Elimination of Htt expression leads to severe motor and behavioral deficits

To investigate the essential roles of Htt in the adult mouse, we ablated Htt expression in cKO mice at 3, 6 and 9 months of age and followed them linearly until end-stage (S2 Fig). TM-treated control (CTL) and cKO mice were injected intraperitoneally with TM for five consecutive days. No tamoxifen-treated (noTM) CTL and cKO mice were injected with vehicle alone as described in Materials and Methods.

**Fig 2:**
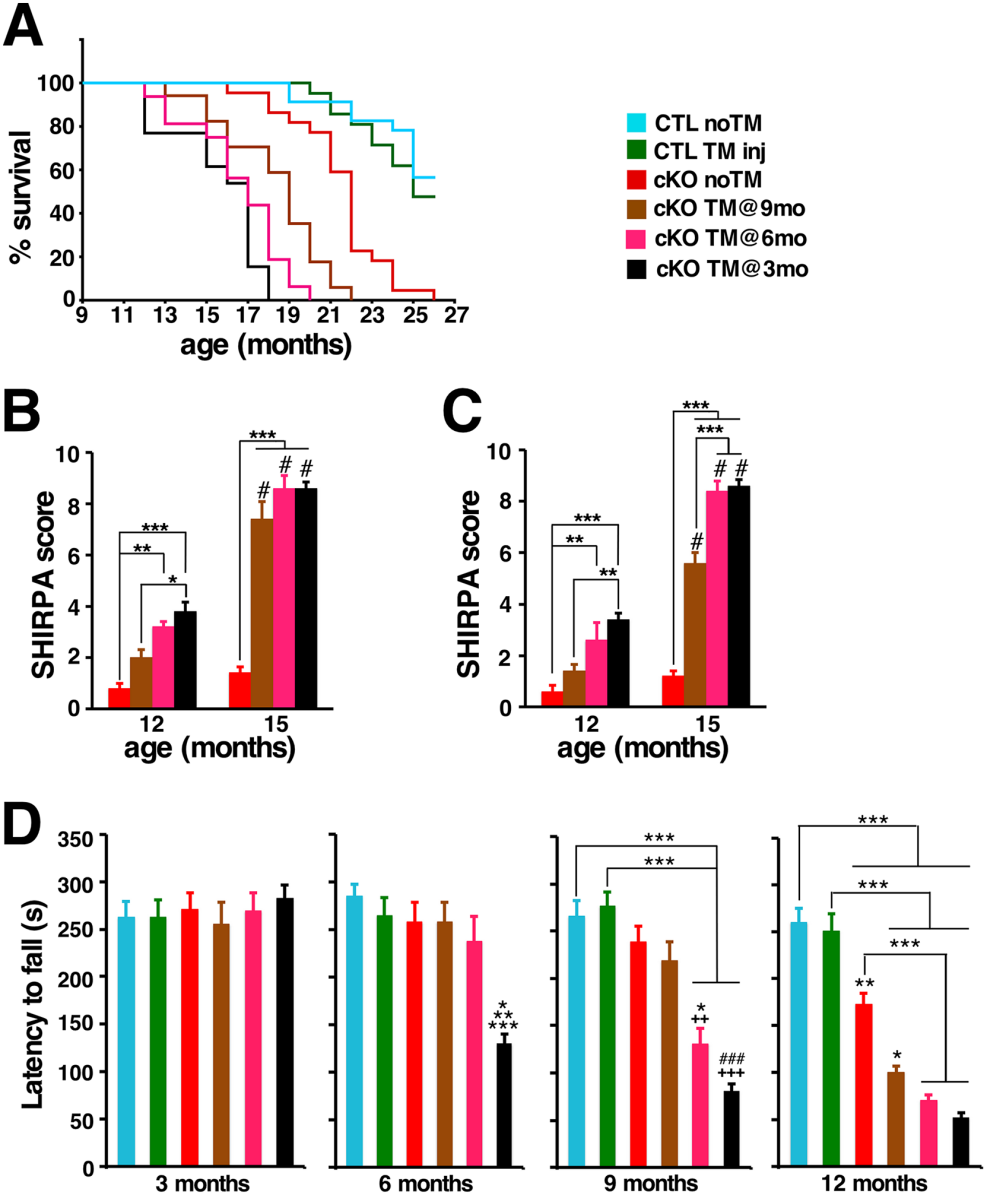
Htt inactivation reduces longevity, and leads to progressive motor and behavioral deficits. (A) Kaplan-Meier survival curves of non-treated controls (CTL noTM, n = 23), controls TM-treated (CTL TM inj, n = 21), non-treated cKO (cKO noTM, n = 22), and cKO TM-treated at 3 months (cKO TM@3mo, n = 13), 6 months (cKO TM@6mo, n = 16) and 9 months of age (cKO TM@9mo, n = 17). P<0.001 between cKO TM@3mo or cKO TM@6mo or cKO TM@9mo and CTL noTM or CTL TM inj. No significant difference between cKO TM@3mo and cKO TM@6mo or CTLnoTM and CTL TM inj. Statistical analysis of survival was performed by log-rank test. Note that Htt elimination results in significant reduction of life-span. (B,C) SHIRPA scores of female (B) and male (C) mice (n = 5 per group and sex). Data from 12 and 15 months of age mice are represented as mean ± SEM. Note that Htt elimination results in behavioral abnormalities in both male and female TM-treated cKO cohorts (*P<0.05; **P<0.01; ***P<0.001 by two-way ANOVA followed by Bonferroni), and that by 15 months of age there is significant progression compared to the 12 months of age time-point (^#^P<0.001, analyzed by Bonferroni). (D) Graphs of latency to fall from an accelerated rotarod for CTL noTM (n = 14); CTL TM inj, (n = 14); cKO noTM (n = 9), cKO TM@9mo, (n = 11), cKO TM@6mo, (n = 8) and cKO TM@3mo (n = 5). Data are expressed as mean ± SEM. Differences between groups were determined by one-way analysis of variance (ANOVA) followed by Bonferroni post hoc test. *P<0.05 versus cKO TM@6mo; **P<0.01 versus CTL TM inj, cKO noTM and cKO TM@9mo; and ***P<0.001 versus CTL noTM for latency to fall at 6 months of age. ^++^P<0.01, ^+++^P<0.001 versus cKO noTM; *P<0.05 versus cKO TM@9mo; ^###^P<0.001 versus cKO TM@9mo; ***P<0.001 for latency to fall at 9 months of age. *P<0.05 versus cKO noTM; **P<0.01 versus CTL TM inj; ***P<0.001 for latency to fall at 12 months of age.

Regardless of the time of Htt elimination, all TM-treated cKO mice developed progressive gait abnormalities, resting tremors, and, by end-stage, decreased locomotor activity. As expected from the deteriorating overall health, survival of TM-treated cKO mice was also compromised (Fig 2A). While 50% of the TM-treated and untreated control mice were alive at 26 months of age, and survival of untreated cKO (cKO noTM) mice majorly declined only after 20 months of age, no cKO mice TM-treated at 3 months (cKO TM@3mo) survived past 18 months of age. Although cKO mice TM-treated at either 6 months or 9 months of age (cKO TM@6mo, cKO TM@9mo) also showed a significantly reduced life-span, cKO mice TM-treated at 9 months survived longer than those treated at 6 months of age.

To evaluate the progression of health and behavioral deficits, we used a modified SHIRPA protocol [20] (see Materials and methods) designed for standardized longitudinal behavioral and gross appearance assessment. Using this protocol, mice of all cohorts were monitored and scored (Fig 2B and 2C; see Materials and methods). Untreated female and male cKO mice did not significantly differ from controls up to 15 months of age. In contrast, 6 months after Htt elimination all TM-treated cKO mice displayed gait abnormalities, resting tremors, and clenching of the hindlimbs on the midline when suspended by the tail. By 15 months of age, all cKO mice TM-treated at either 3 or 6 months of age displayed significant worsening of the initial phenotypes, kyphosis, and showed a pronounced weakness in the hindlimbs, which affected their ability to walk or even to stand up (S1 Movie, S2 Movie). Progression of the same phenotypic features was observed in cKO mice TM-treated at 9 months of age, although with a significant delay.

To further examine the onset and progression of motor dysfunction, we performed rotarod performance tests on untreated and TM-treated cKO mice and controls. For this assessment, mice were tested once a week on an accelerating rotarod, starting at 5 weeks of age (see Materials and methods). Surprisingly, TM-treated cKO mice showed a significant impairment on rotarod performance already one month after Htt elimination (S3 Fig). Rotarod performance then declined steeply thereafter in TM-treated cKO cohorts so that 6 months after Htt elimination all TM-treated cKO cohorts had an average latency to fall about 30% their pre-TM-treated values (Fig 2D, S3 Fig).

In parallel, weight gain was also monitored. In all cohorts, intraperitoneal TM administration resulted in a temporary 10% body weight loss in all genotypes. In male cohorts, TM-treated and untreated controls as well as untreated cKO mice displayed a similar weight gain rate, gaining an average of 12.0 to 14.0g over a 12-month period (from 3 months to 15 months of age). In contrast, the body weight curve of TM-treated cKO male mice followed a biphasic mode, displaying a similar weight gain rate relative to untreated or TM-treated cohorts over the first months after Htt elimination, while about 6 months after Htt elimination weight gain rate became negative (S4A Fig, S1–S3 Tables). In female cohorts, the average weight gain of untreated controls for the same 12-month period was about 14.0 g. Surprisingly, while tamoxifen administration did not impact weight gain in control males, it did have an adverse and irreversible effect in control females, significantly reducing their weight gain rate over time compared to untreated control females. TM-induced Htt elimination in female mice also followed the same biphasic mode described above for the TM-treated cKO male mice (S4B Fig, and S4–S6 Tables).

In the course of our analyses we also noted that a significant fraction of TM-treated cKO mice developed several other abnormalities. Corneal opacity and thickening of the cornea, usually seen in aged mice [21, 22], was observed by visual inspection in about 33% (n = 14/42) of TM-treated cKO mice between the ages of 9 and 18 months (S5 Fig), while it occurred in only about 6% (n = 3/54) of control mice aged 17–23 months. Also, prolapsed rectum was observed in about 50% of TM-treated cKO females and 20% of males between the ages of 12 and 19 months (S6A Fig), while only 10% of control females of the same age range displayed this feature. In addition, urinary retention, leading to overdistension of the urinary bladder was observed in 70% of TM-treated cKO males at the time of sacrifice, but was never observed in control males or in TM-treated cKO females. As expected, global loss of Htt expression also resulted in testicular atrophy [16, 23], which could be already observed 3 months after Htt elimination (S6B Fig, S7 Table), but did not grossly affect other peripheral organs (S7 Fig and S8 Table).

### Htt elimination leads to progressive brain atrophy and widespread reactive gliosis

Since HD phenotypic features are caused primarily by neurodegeneration of the central nervous system, Htt is expressed at high levels in the adult brain and Htt elimination in the developing and early postnatal mouse brain causes progressive neurodegeneration [16, 24, 25], it was fundamental to assess if Htt elimination in the adult mouse brain caused any pathological changes.

**Fig 3:**
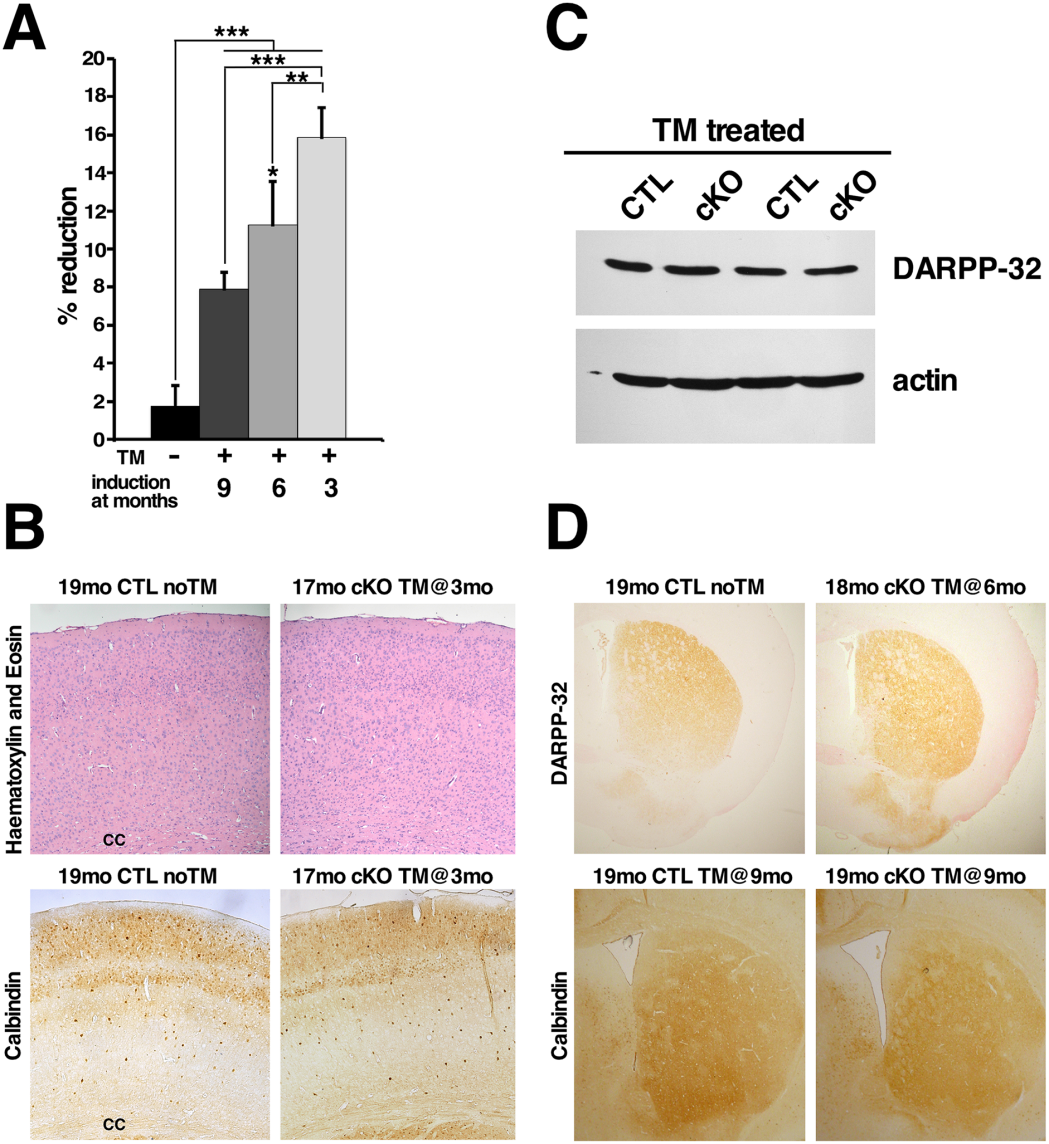
Loss of Htt causes brain atrophy but does not lead to overt pathological changes in cortex or striatum. (A) Bar graph represents the percentage of brain weight reduction of 17–19 months of age non-treated (n = 6), and TM-treated CreER; *Hdh*^*flox/-*^ (n = 5 for each treatment group) mice (analyzed by ANOVA followed by Bonferroni test, *P<0.05 versus 9 months; **P<0.01, ***P<0.001). Results are presented as percentage of brain weight reduction compared to littermates/aged-matched TM-treated control brains. Values represent mean ± SD. (B) Representative H&E and calbindin-stained coronal sections of brains 19mo CTL noTM and 17mo cKO TM@3mo. Note that the thickness, cell density, and distribution of calbindin-positive neurons is similar in the cortex of controls and mice lacking Htt, even 14 months after Htt elimination. cc = corpus callosum. (C) Representative western blot of DARPP-32 protein analysis in 10 month-old brains from controls (CTL) and cKO mice TM-treated at 6 months of age. Antibody against actin was used as internal control for loading. Note that DARPP-32 levels are unchanged 4 months after Htt elimination. (D) Representative coronal sections of brains from 19mo CTL noTM and 18mo cKO TM@6mo immunostained for DARPP-32, and 19mo CTL TM@9mo and 19mo cKO TM@9mo immunostained for Calbindin. Note that the intensity and distribution of DARPP-32 and Calbindin in the striatum is similar between controls and TM-treated cKO mice months after Htt elimination.

Gross examination of TM-treated cKO brains at different time-points did not reveal any obvious abnormalities, however brain weight loss was noted in all TM-treated cKO mice. The extent of brain weight loss was dependent on the time elapsed since Htt elimination, and hence at 18 months of age it was more pronounced in cKO mice that were TM-treated at 3 months of age compared to age-matched mice treated at 6 or 9 months of age (Fig 3A).

Given that cortex and striatum are the most vulnerable brain regions in HD, and are therefore the primary targets for therapeutic strategies aiming at reducing or eliminating Htt expression, we first assessed the impact of Htt loss in these two brain regions. In the cortex, H&E-staining and Calbindin D28K immunohistochemistry on coronal brain sections of 17 month-old cKO mice TM-treated at 3 months of age appeared normal (Fig 3B). Closer investigation did not reveal significant differences in cortex thickness or calbindin neuronal numbers compared to controls (Fig 3B; S9 Table). These findings indicate that even 14 months after Htt elimination there is no clear evidence of overt cell loss in the cortex.

**Fig 4:**
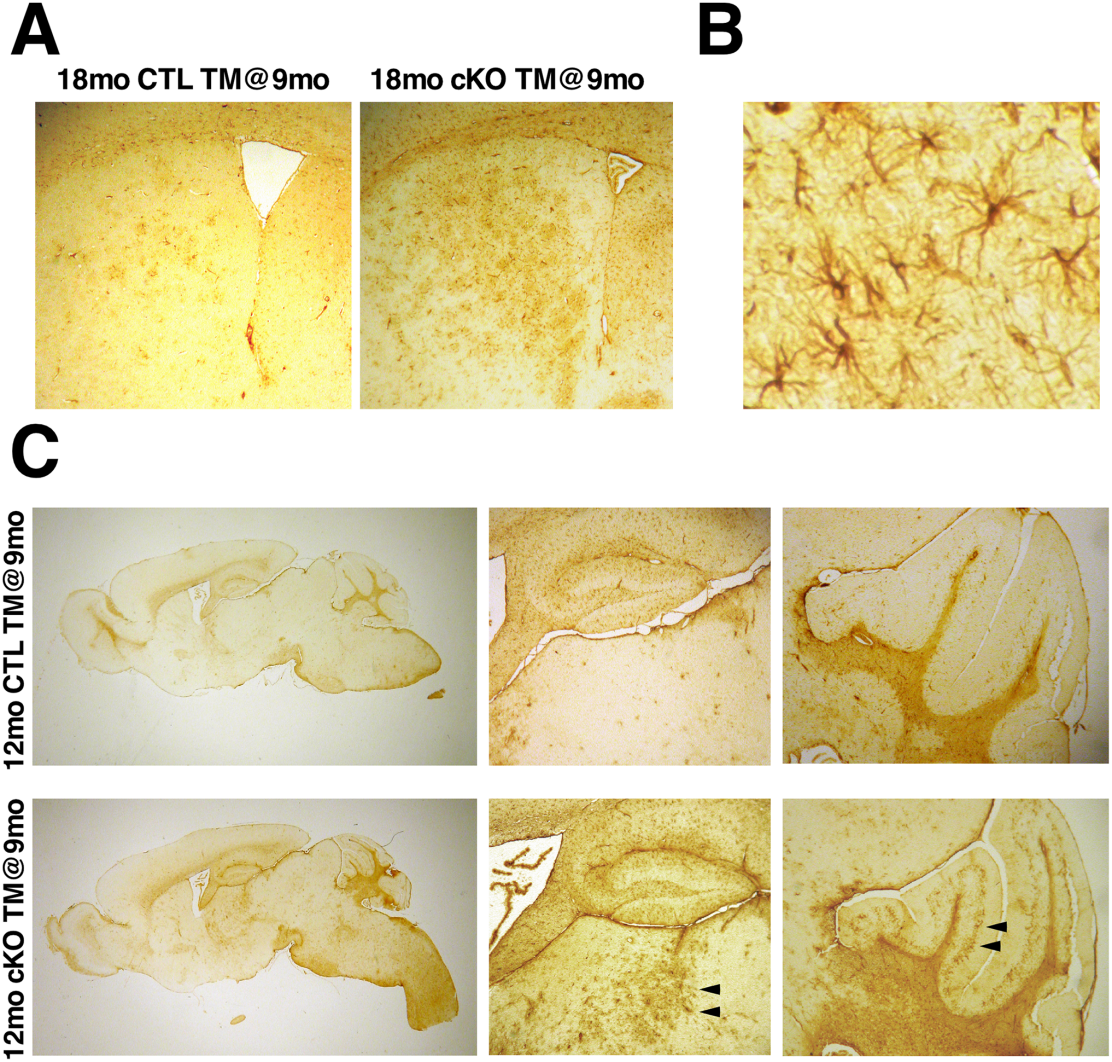
Htt elimination results in widespread reactive gliosis. (A) Representative GFAP immunostained coronal sections through the forebrain of 18mo CTL TM@9mo and 18mo cKO TM@9mo. Note the extensive reactive gliosis in the dorsal striatum 9 months after Htt elimination. (B) High magnification image showing the characteristic morphology of GFAP-positive reactive astrocytes in the brain of 18mo cKO TM@9mo mouse. (C) Representative sagittal sections through the brains of 12mo CTL TM@9mo and 12mo cKO TM@9mo immunostained for GFAP. Note that although there are no signs of reactive gliosis in the striatum at this time-point, extensive GFAP staining is observed in the mediodorsal thalamus and cerebellum of 12mo cKO TM@9mo mice three months after Htt elimination (arrowheads).

To assess if Htt elimination results in neuronal cell loss or dysfunction in the striatum, we first analyzed expression of DARPP-32, a protein that is highly and specifically expressed in medium spiny neurons, which account for 90–95% of neuronal cells in the striatum [5, 26]. Western blot analyses on brain protein extracts four months after Htt elimination did not reveal changes in DARPP-32 levels in cKO mice compared to controls (Fig 3C). Immunohistochemistry for DARPP-32 on coronal brain sections of 18 month-old cKO mice TM-injected at 6 (that is 12 months after Htt elimination) confirmed that no significant changes in DARPP-32 expression or noticeable cell loss occurred in the striatum even after long-term Htt loss (Fig 3D; S10 Table). Immunohistochemistry for Calbindin D28K (highly expressed in medium spiny neurons) also did not reveal significant differences (Fig 3D, S10 Table). Altogether, these results indicate that survival of medium spiny neurons is not significantly affected after long-term Htt elimination. Notably though, GFAP immunohistochemistry revealed the presence of extensive reactive gliosis in the dorsal striatum 9 months after Htt elimination (Fig 4A and 4B, S8A Fig), indicating that although long-term Htt elimination does not result in overt neuronal cell death in the striatum, it does lead to reactive astrocytosis.

To determine whether Htt loss affected other brain regions, we assessed for the presence of reactive gliosis by immunohistochemistry for GFAP in sagittal brain sections of TM-treated cKO mice at different time-points. Strikingly, extensive reactive gliosis was observed in the thalamus and granular cell layer of the cerebellum already 3 months after Htt elimination (Fig 4C, S8B Fig) at a time when no signs of reactive gliosis were yet observed in cortex and striatum.

### Htt elimination results in bilateral thalamic calcification

**Fig 5:**
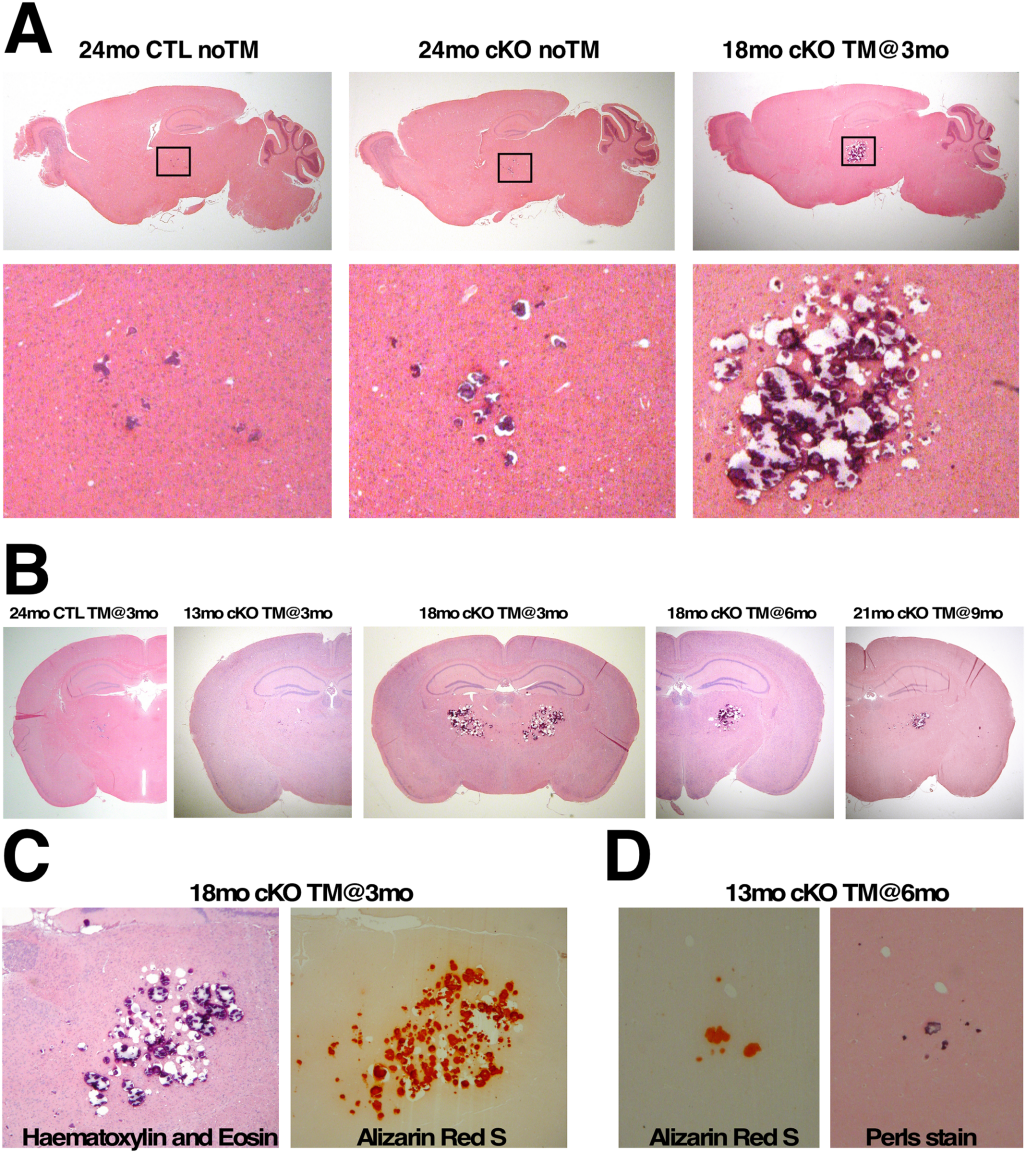
Progressive brain thalamic calcifications in mice lacking htt. (A) H&E-stained sagittal brain sections of 24mo CTL noTM and 24mo cKO noTM mice, and of 18mo cKO TM@3mo. Note that although discrete thalamic lesions can be observed in aged CTL and untreated cKO mice, Htt elimination causes extensively large thalamic lesions (insets). (B) H&E-stained coronal brain sections of 24mo CTL TM@3mo, 13mo cKO TM@3mo, 18mo cKO TM@3mo, 18mo cKO TM@6mo, and 21mo cKO TM@9mo. Images in (A) and (B) are representative photographs of thalamic lesions at their largest dimensions. Note that the size of the thalamic lesions depend on both, the time elapsed since Htt elimination and the age at which htt was eliminated. (C) Adjacent brain sections from 18mo cKO TM@3mo stained with H&E or Alizarin Red S show the presence of calcium in thalamic lesions. (D) Adjacent brain sections from 13mo cKO TM@6mo stained with Alizarin Red S or Perls’ showing co-localization of calcium and iron in the lesions.

During the course of our analyses we also noticed the presence of large solid deposits located symmetrically in the thalamic areas in the brains of 18 month-old TM-treated cKO mice. These deposits caused nicking of the microtome blade and ripping of the sections during sectioning, and stained strongly with haematoxylin (Fig 5A). To determine the time-course of formation and progression of the thalamic lesions, we performed histological analyses on sagittal and coronal sections of control and of cKO mice at different time-points after Htt elimination. Our results indicate that small sparse thalamic lesions are present in the brains of aged non-TM-treated cKO and control mice (Fig 5A and 5B), but are rarely observed prior to 18 months of age (seen in about 7%, n = 2/29). In contrast, elimination of Htt at 3 or 6 months of age leads to the appearance of small thalamic lesions as early as 13 months of age (Fig 5B). These lesions, consistently observed between 15 and 18 months of age in all TM-treated cKO mice (n = 12), progress gradually, occupying a large portion of the mediodorsal thalamus by 18 months of age (Fig 5B). Similarly, large lesions in the mediodorsal thalamus are also consistently seen in the brains of 18–21 month-old cKO mice TM-treated at 9 months of age (n = 8), although not as pronounced (Fig 5B). Significantly, these lesions were confined to the thalamus at all time-points examined.

To verify the nature of the thalamic lesions, adjacent coronal sections spanning the thalamus were stained with Alizarin Red to assay for the presence of calcium and with Perls’ stain to assay for the presence of iron. As shown in Fig 5C and 5D thalamic lesions stained strongly with Alizarin Red and also displayed positive Perls’ stain, indicating the presence of both calcium and iron.

Hyperphosphatemia, as seen in diseases including hypoparathyroidism and pseudohypoparathyrodism is associated with intracranial as well as renal calcifications in humans [27] and consistently lead to prominent renal calcifications in mice [28–30]. Alizarin red staining of kidneys from 15mo cKO TM@3mo mice however did not reveal the presence of renal calcifications (S9 Fig), indicating therefore that the brain thalamic calcifications are not due to hyperphosphatemia.

### Elimination of Htt leads to altered brain iron homeostasis

Intracranial calcifications containing iron are reminiscent of cerebrovascular ferrocalcinosis [31–33], suggesting that a similar mechanism may underlie the formation of calcifications due to Htt elimination. Since defective iron metabolism appears to contribute to the formation of intracranial calcifications in cerebrovascular ferrocalcinosis [32, 34, 35] and Htt has been implicated in iron transport in mouse and zebrafish embryos [36, 37], as well as in mouse embryonic stem cells [38], we investigated whether altered brain iron homeostasis could be a contributing factor to the progressive thalamic calcification.

Iron homeostasis is a process that is tightly controlled in the brain, via regulation of key molecules involved in iron transport and storage. Regulation of several of these molecules occurs at the translational level. For instance, when iron levels are low, iron responsive proteins (IRPS) mediate the downregulation of translation of the iron storage protein ferritin (Ft), while translation of the main iron import protein transferrin receptor (Tfr) is upregulated, as a compensatory mechanism to increase iron uptake in the cells. Concomitantly, the membrane-bound ferroportin (Fpn1), the main mammalian iron export protein, is internalized and degraded preventing further iron loss from the cells. The opposite is observed when intracellular iron levels are elevated [39, 40].

**Fig 6:**
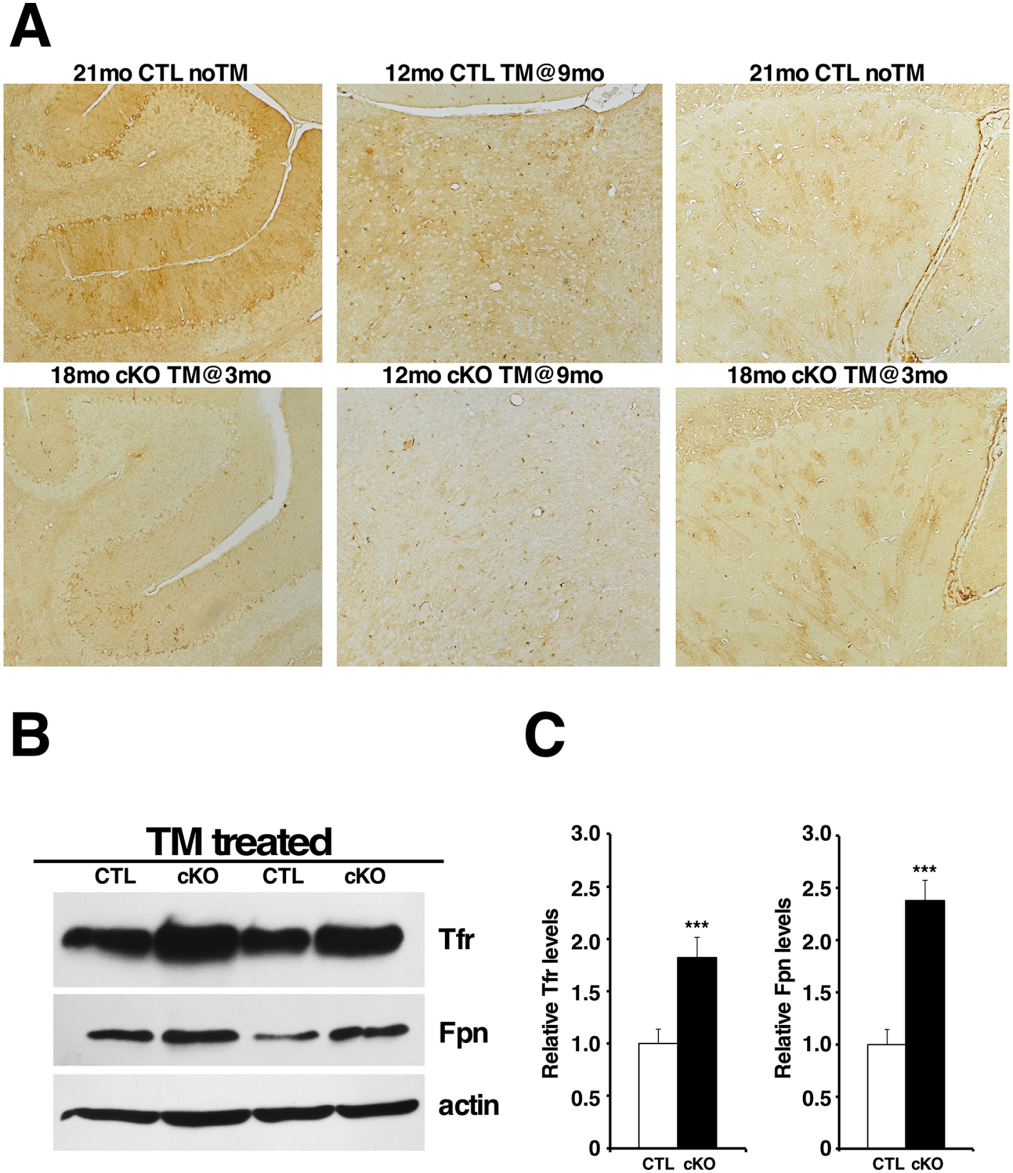
Htt elimination disrupts brain iron homeostasis. (A) Representative photographs of immunohistochemical staining for ferritin light chain (Ft). Left panels: cerebella of 21mo CTL noTM and 18mo cKO TM@3mo; middle panels: thalami of 12mo CTL TM@9mo and 12mo cKO TM@9mo; right panels: striata of 21mo CTL noTM and 18mo cKO TM@3mo. Note that Ft levels are already extremely reduced in the thalamus 3 months after Htt elimination (middle panels), and that Ft expression is practically abolished in the cerebellum in the absence of Htt (left panels). (B) Representative western blots of Tfr and Fpn protein expression in 10 month-old brains from CTL and cKO mice TM-treated at 6 months of age. Antibody against actin was used as internal control for loading. Note that Tfr and Fpn levels are increased in TM-treated cKO compared to controls. (C) Quantification of Tfr and Fpn expression levels. Western blots of total protein extracts from 10 month-old brains CTL (n = 5) and cKO (n = 5) TM-treated at 6 months of age were probed with anti-Tfr or anti-Fpn, stripped and re-probed with anti-actin antibody. Bands intensities were quantitated using Image J. Tfr and Fpn levels were normalized over actin levels. Values represent mean relative to controls ± SD (***P<0.001, Student’s t-test).

Immunohistochemistry for ferritin light chain (Ft) showed that already three months after Htt elimination, brain Ft levels are significantly reduced in TM-treated cKO mice compared to controls, especially in the thalamus and cerebellum, and remain low throughout their life-span (Fig 6A). Consistent with the reduction in Ft levels, we found that brain Tfr levels were significantly increased in TM-treated cKO mice after Htt elimination (Fig 6B and 6C). Unexpectedly, we found that instead of downregulated, Fpn levels are significantly upregulated in the brain when Htt expression is abolished (Fig 6B and 6C).

**Fig 7:**
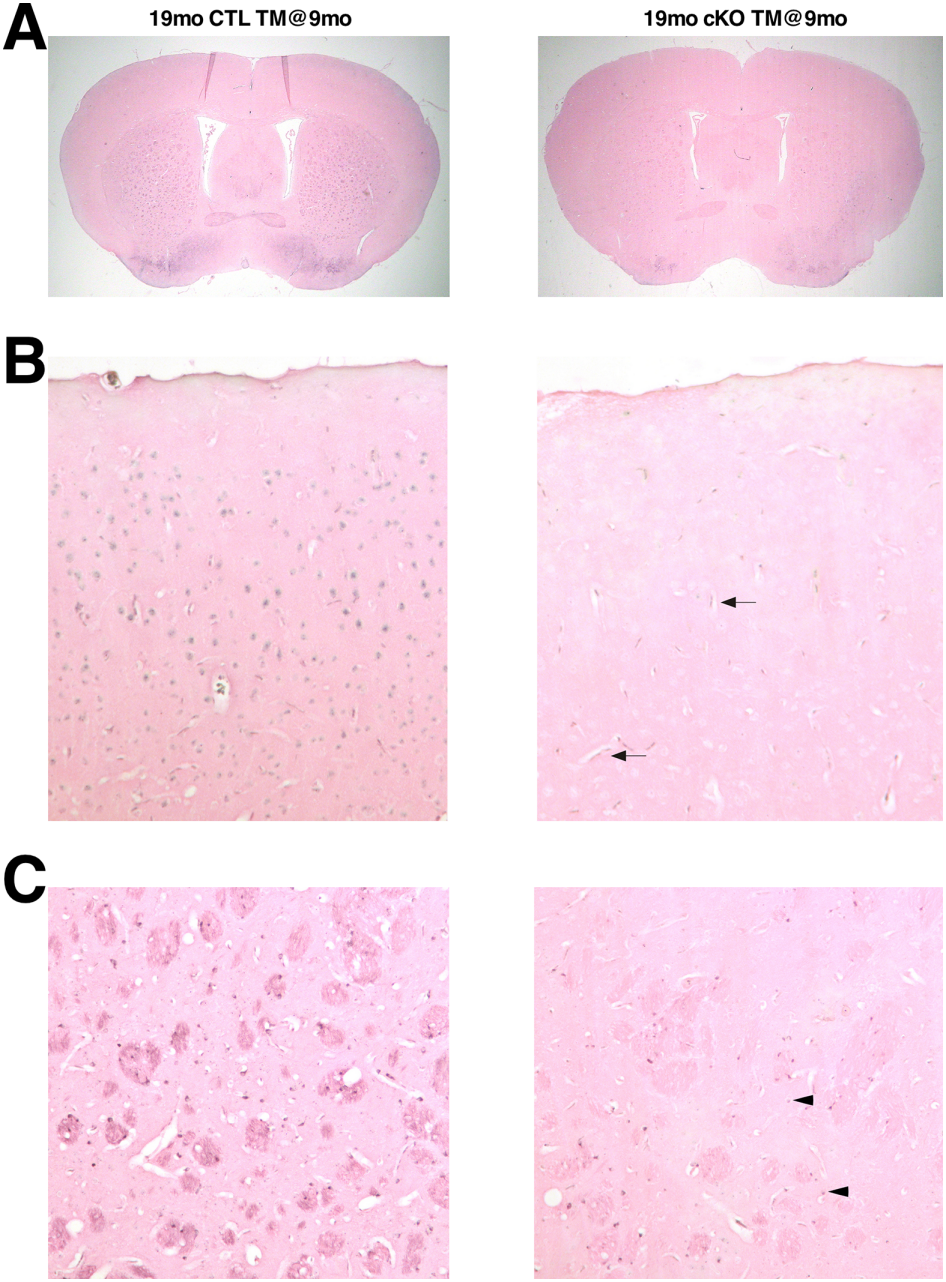
Reduced brain ferric iron content in mice lacking Htt. Representative images of Perls’ stained (brown signal) coronal forebrain sections of 19mo CTL TM@9mo and 19mo cKO TM@9mo. (A) Low magnification of forebrain coronal sections; (B) high magnification of cerebral cortex; (C) high magnification of striatum. Note the near absence of iron staining in neuronal cells of TM-treated cKO brains (B, C), although iron staining is visible in vessels (arrows) and oligodendrocytes (arrowheads).

The altered expression levels of the iron regulatory proteins Tfr and Ft suggest that iron levels are reduced in the brain in the absence of Htt. To verify this premise, we assessed brain iron cellular distribution and relative levels by Perls’ iron staining in TM-treated CTL (n = 5) and cKO (n = 5) mice. Although Perls’ iron staining could be observed in oligodendrocytes and vessels, ferric iron levels were overall significantly reduced throughout the brains of TM-treated cKO mice, including in the striatum and cortical neuronal cells (Fig 7, S10 Fig). Together these findings indicate that loss of Htt leads to chronic iron depletion in the brain.

Since with our experimental design Htt is globally eliminated, the reduced iron content in the brains of mice lacking Htt could potentially be due to either altered brain iron homeostasis or secondary to a systemic deficit. Maintenance of systemic iron homeostasis is largely ensured by iron recycling in splenic and hepatic macrophages. In addition, the liver is majorly responsible for either storing excess iron in ferritin or mobilizing it back to the circulation, depending on the metabolic requirements [40–42]. Perls’ staining of liver and spleen paraffin sections from TM-treated cKO mice however did not reveal any differences in iron content and distribution compared to CTL (S11 Fig), indicating that neither iron storage nor recycling are impaired in the absence of Htt, and that the reduced brain iron content is likely due to a need of Htt for maintenance of brain iron homeostasis.

## Discussion

Using the Cre-loxP system of recombination, here we show that elimination of Htt in the adult mouse at 3, 6 or 9 months of age consistently leads to progressive motor and behavioral decline, reduced life-span and extensive neuropathology. Significantly, Htt elimination causes the same time-dependent defects regardless of the stage at which the animals were treated (S12 Fig).

Previous work by different research groups indicates that short-term reduction of Htt expression is well tolerated in the striatum of mice and macaques [9–12]. Likewise, short-term elimination of Htt in adult mouse neuronal cells does not result in overt pathology in the striatum [43]. Consistent with these findings, we have found that in the adult mouse brain complete elimination of Htt expression does not lead to obvious pathology in the striatum (Fig 3C, 3D and S10 Table), suggesting that, in the mouse, even long-term Htt elimination does not impair medium spiny neuron survival. Likewise, no gross pathology was observed in the cortex after prolonged Htt elimination (Fig 3B and S9 Table). Together, these observations indicate that long-term elimination of Htt is well tolerated in the adult cortex and striatum, the regions that are primarily affected in HD, and highlight a fundamental difference between the impact of loss of normal huntingtin compared to a gain of function due to expression of mutant huntingtin.

Using a similar approach to the one described in this manuscript, Wang and collaborators globally eliminated Htt in *Hdh*^*flox/flox*^ mice at 2, 4 and 8 months of age [43]. In contrast to our results, they did not observe decline in rotarod performance even 11 months after global Htt elimination, nor were any behavioral abnormalities reported. The same group also eliminated Htt in neurons of adult mice using tamoxifen-inducible neuron-specific Cre lines with no observed histopathological changes. In face value, these results appear to suggest that Htt elimination in the adult mouse on a hemizygous background (starting with 50% Htt levels as used in our study) might be more deleterious than Htt elimination on a *Hdh*^*flox/flox*^ mouse that expresses normal levels before cre-mediated recombination as in Wang et al., [43]. An alternative explanation is that differences in genetic backgrounds might account for these discrepancies since Pla and collaborators showed that elimination of Htt specifically in cortical and hippocampal neurons in the adult *Hdh*^*flox/flox*^ mouse leads to behavioral abnormalities, altered BDNF signaling, decreased survival and abnormal dendritic arborization of hippocampal newborn neurons already 6 months after Htt elimination [44].

The most striking and unexpected neuropathological finding was that elimination of Htt in the adult mouse brain resulted in progressive bilateral thalamic calcification, encompassing mainly the mediodorsal thalamus (Fig 5A and 5B). Brain calcification, especially in the basal ganglia, is a relatively common radiologic finding in humans, observed in 1–3% in young individuals, and considerably prevalent in the ageing population [45–47]. In ageing mice, discrete small thalamic calcifications are also commonly found [48–51] (Fig 5A and 5B). However, the extensive bilateral thalamic calcifications that we observed in mice lacking Htt are more reminiscent of what is observed in mouse models of a series of rare diseases known as Familial Idiopathic Basal Ganglia Calcifications (FIBGC), cerebroferrocalcinosis/Fahr disease or Primary Familial Brain Calcification (PFBC). PFBC is a genetically heterogeneous neurological condition characterized by symmetrical bilateral calcifications prominently in the basal ganglia, and to a lesser extent in cerebellum, thalamus, and brain-stem [52–56]. It is important to note that mouse models of PFBC display bilateral calcifications predominantly in the thalamus, and to a lesser extent in other brain regions [54, 56]. It is likely therefore that the differences in spatial distribution of the calcifications between mice and humans reflect species-specific characteristics. Histochemical analysis of basal ganglia calcifications in PFBC/FIBGC/Fahr diseases has consistently revealed the presence of iron in the lesions, usually preceding the accumulation of calcium [47]. Similarly, our analyses revealed the presence of iron and calcium deposits in the thalamus of mice lacking Htt already at the early stages of lesion formation (Fig 5D).

Extensive reactive gliosis was observed in the thalamus and cerebellum already three months after Htt elimination (Fig 4), several months before the appearance of the lesions in the thalamus, suggesting that a chronic condition precedes the formation of the thalamic lesions. Significantly though, reactive gliosis in the dorsal striatum is not observed at this stage, but instead coincides with the appearance and progression of the thalamic lesions, suggesting a causal relationship. Since the dorsal striatum receives projections from the thalamus, it is possible that the relatively late reactive gliosis in the striatum might actually be secondary to thalamic injury [57, 58].

The presence of iron in the thalamic lesions, as well as the implication of iron imbalance PFBC/FIBGC/Fahr disease [32, 34, 35], led us to investigate the role of Htt in brain iron homeostasis. Previous work from us and others has implicated Htt in iron transport and iron homeostasis in embryogenesis, as well as in cultured mouse embryonic stem cells [36–38]. Our results show, for the first time, that Htt plays an essential role in adult brain iron homeostasis, and that loss of Htt results in chronic reduction of iron levels throughout the adult brain (Figs 6 and 7). Iron requirement in the brain is relatively high compared to other tissues, due to high energy demands. In the brain, iron is essential for several processes, including ATP production, synthesis of neurotransmitters, axonal transport, and myelin synthesis. In fact, iron deficiency in the central nervous system has been shown to lead to cognitive and motor function deficits [59–61], and could therefore also explain the motor and behavioral decline observed in mice lacking Htt, as well as the extensive reactive gliosis observed in diverse brain regions. Importantly, altered brain iron homeostasis in mice lacking htt precedes the onset of thalamic calcifications by several months substantiating the possibility that iron imbalance might be a primary cause for the thalamic lesions.

The mechanisms by which Htt loss leads to iron dyshomeostasis are still not fully clarified. It has been proposed that intracellular iron trafficking and/or release from endocytic vesicles might be impaired in the absence of Htt. This hypothesis is based on the observations that Tfr accumulates in the cytoplasm of Hdh null embryonic stem cells [37, 38] and that Htt is required for intracellular vesicular trafficking of Rab11 [62, 63], a GTPase shown to be involved in Tfr recycling in epithelial cells [64]. The mechanisms involved in Tfr recycling however appear to vary depending on cell type. For instance, in primed PC12 cells Rab11 and Tfr do not co-localize in the same recycling endosomes [65] suggesting that at least in some neuronal cell populations Tfr recycling may not depend on Rab11. Although impaired Htt-dependent Tfr recycling could potentially explain the relative increase in Tfr protein levels and concomitant decrease in ferritin levels and neuronal intracellular iron that we observed in adult brains lacking Htt, the observation that ferroportin levels are concomitantly elevated raise the possibility that an increase in ferroportin levels may actually precede iron loss. This hypothesis is supported by the observation that overexpression of Fpn in several cellular systems is sufficient to decrease intracellular iron levels and secondarily result in decrease in Ft and increase in Tfr levels [66, 67].

In summary, our studies show that Htt is an essential protein in the adult mouse, and in particular in the brain, since Htt elimination leads to thalamic calcifications and altered brain iron homeostasis. Although the global genetic elimination of Htt described herein differs from the clinical trial paradigm, in which the aim is to lower (and not eliminate) Htt expression specifically in the brain in the context of HD, the extensive behavioral and pathological deficits that we observed in our gene-inactivation paradigm still caution against the use of non-allele specific gene expression silencing strategies, and indicate that therapies targeting specifically the mutant Htt allele in HD patients [68–71] might represent a safer alternative. On a positive note our results also showed that long-term Htt elimination is well tolerated in the adult cortex and striatum, the regions that are primarily affected in HD and therefore constitute the main targets for therapy.

## Materials and methods

### Ethics statement

All animal experiments were performed according to protocols with #1154 and #2059 approved by the Institutional Animal Care and Use Committee (IACUC) at the University of Tennessee Health Science Center (UTHSC). Euthanasia was performed under CO_2_ anesthesia followed by cervical dislocation or isoflurane anesthesia followed by cervical dislocation.

### Animals

Experimental CreER^™^; *Hdh*^*flox/-*^ mice, expressing htt protein at 50% that of wild-type levels, and control littermates were generated through a series of genetic crosses. Briefly, *Hdh*^*+/-*^ mice [19] (Mouse Genome informatics (MGI) designation Htt^tm1Szi^) were crossed with CAGG-Cre-ER^™^ mice [18] (purchased from the Jackson Laboratory with strain name: *B6*.Cg-Tg(CAG-cre/Esr1*)5Amc/J and stock number: 004682, thereafter referred as CreER), and the resulting CreER; *Hdh*^*+/-*^ progeny was then crossed with *Hdh*^*flox/flox*^ mice [16] (MGI designation Htt^tm2Szi^). CreER; *Hdh*^*flox/-*^ (thereafter referred as cKO) mice were obtained at the expected 1/4 Mendelian ratio. All mouse strains were on C57Bl/6 background.

Mice (almost equal number for each gender) were divided into the following four groups:

1. no-TM-injected (noTM) *Hdh*^*flox/+*^ and *Hdh*^*flox/-*^. This group represents the noTM injected control (CTL noTM).
2. no-TM-injected CreER; *Hdh*^*flox/-*^ (cKO noTM). This group represents the noTM injected cKO and provides a control for leaky cre recombination.
3. tamoxifen injected *Hdh*^*flox/+*^ and *Hdh*^*flox/-*^ (CTL TM inj) This group represents the tamoxifen-treated control mice and provides information about the potential side effects of tamoxifen treatment on the overall health and behavior of the mouse and also provides an indication of any effects on huntingtin levels from tamoxifen treatments.
4. tamoxifen injected CreER; *Hdh*^*flox/-*^ (cKO TM inj) This group represents the treated experimental group and provides the experimental mice for our studies on the effects of loss of Htt.

For all experiments, tamoxifen (abbreviated as TM, Sigma, T-5648) was prepared at a concentration of 10mg/ml in corn oil containing 10% ethanol. Solubilization of tamoxifen was achieved by incubation at 37°C for 4–6 hrs, vortexing every 30 min. Unless stated otherwise, tamoxifen, 2.0 mg/26g body weight, was injected intraperitoneally for 5 consecutive days to the tamoxifen-treated experimental and tamoxifen-treated control groups (“c” and “d”). No-TM-injected (noTM) CTL and cKO groups (“a” and “b”) were injected with vehicle alone. Body weights of males and females (untreated and TM-treated) were recorded weekly starting at 5 weeks of age.

In practical terms, 4 cohorts of mice were followed and analyzed: cohort #1 included the cKO noTM and CTL noTM groups (“a” and “b”) (S2 Fig), while tamoxifen-treated experimental cKO and tamoxifen-treated CTL groups (“c” and “d”) were divided into three cohorts: cohort #2 (TM administration at 3 months of age), cohort #3 (TM administration at 6 months of age), and cohort #4 (TM administration at 9 months of age) (S2 Fig).

### PCR assays for genotyping

For routine genotyping of progeny, genomic DNA was prepared from tail biopsies as described previously [72], and PCR amplification reactions were carried in the following conditions: 30 cycles consisting of 45 sec denaturation at 94°C, 45 sec annealing at 61°C, and 1 min extension at 72°C.

The Hdh null allele was detected using the primers Neo 3 (Neo reverse) 5’- AGAGCAGCCGATTGTCTGTTGT-3’ and Neo 6 (MC1 promoter forward) 5’- AACACCGAGCGACCCTGCAG-3’, which generate a 130 bp product. The cre transgene was detected using the primers Cre 1 (Cre forward): 5´-CTGCCACGACCAAGTGACAGC-3´ and Cre 2 (Cre reverse): 5´-CTTCTCTACACCTGCGGTGCT-3´ to generate a 325 bp product corresponding to a portion of the cre coding region as described previously [73].

The Hdh floxed allele was detected using the primers pgk (pgk promoter reverse): 5’- AGCGCATGCTCCAGACTGCC-3’ and Hdhpr13 (Hdh promoter forward): 5’-CTGTCTGGAGGTGATCCATGC-3’, which generate a 210 bp PCR product. The Hdh WT allele was detected using the primers Hdhpr13 and Hdhwt10 5’- AGGGGACATAGAATCTAAAGGCT-3’, which generate a 210 bp product.

### Assessment of cre-mediated recombination at the DNA level

Brains were dissected and genomic DNA from cortex, striatum, and cerebellum of 4 month-old cKO TM-treated at 3 months were submitted to semi-quantitative PCR amplification using oligos that differentially amplify the Hdhflox (unrecombined) and HdhΔflox (recombined) alleles. For each PCR reaction the following oligos were used: Pgk, Hdhpr13 to generate a PCR product of 210 bp for the unrecombined allele and Hdhrec9: 5’- AGGCCAGAGACCCCGCAAGA-3’ to generate a PCR product of 220 bp for the recombined allele. PCR reactions products were fractionated in 2% agarose gels, and stained with ethidium bromide. DNAs from different organs were analyzed as above. Images were captured using a Kodak in vivo imaging system F pro, and band intensities (unrecombined and recombined) were analyzed using Kodak Molecular Imaging system. The percentage of recombination was calculated by dividing the value of the recombined allele band by the sum of the values of the unrecombined and recombined bands.

### Assessment of Hdh expression by RT-PCR

Brains were dissected and total RNA was isolated from brain or cortex and striatum of 4 month-old cKO, non-TM-treated cKO and 4 month-old TM-treated cKO mice (one month after TM administration) using Trizol reagent (GIBCO-BRL), according to the manufacturer’s instructions. For reverse transcription, samples were first treated with RQ1 DNAse (to remove DNA contaminants), and 1μg total RQ1 DNAse-treated RNA was annealed with random hexamers, and first strand cDNA synthesis was carried out using Transcriptor 1^st^ strand synthesis kit (Roche). For assessment of Htt gene expression, 1μl of each RT reaction (equivalent to 50 ng of starting mRNA) was used for semi-quantitative PCR amplification. All PCR reactions were carried out in the same conditions: 45 sec denaturation at 94°C, 45 sec annealing at 61°C, and 1 min extension at 72°C, with the primers Hdh11 (forward) 5´-TGTGGATATCTGGAATCCTCGC-3´ and Hdh12 (reverse) 5´- GAACGTATGCTGCTGTTCACTC-3´ to generate a 300 bp product. Hdh11 is located in exon 39 while Hdh12 is in exon 40 of *Mus musculus huntingtin (Htt)*, *mRNA GenBank NCBI Reference Sequence*: *NM_010414*.*3*, and the amplified product spans positions 5122–5420 of the above-mentioned sequence.

For internal control, 18S rRNA was amplified by PCR consisting of 45 sec denaturation at 94°C, 45 sec annealing at 61°C, and 1 min extension at 72°C, using the primers 18Sfor 5’-GGTGGTGGTGCATGGCCGTT-3’ and 18Srev 5’- GCAGCCCCGGACATCTAAGG-3’ which amplify a 200 bp product.

PCR reactions products were fractionated in 2% agarose gels, and stained with ethidium bromide. Images were captured using a Kodak in vivo imaging system F pro, and band intensities were analyzed using Kodak Molecular Imaging system.

### Western blot analysis

Protein extracts were obtained by homogenizing tissue in RIPA buffer (150mM NaCl, 50mM Tris pH 8.0, 1% NP-40, 0.1% SDS, 0.5% Sodium Deoxycholate) containing protease inhibitor cocktail (Roche). Insoluble debris was discarded after centrifugation, and protein concentration was determined by the Bradford assay (Bio-Rad). Approximately 50 μg of protein was separated by SDS-PAGE and transferred into nitrocellulose membranes. Membranes were blocked in 5% non-fat milk for 1 hr at room temperature and incubated overnight at 4°C with mouse monoclonal anti-huntingtin (MAB 2166, Chemicon); rabbit polyclonal anti-DARRP-32 (AB 1656, Chemicon); mouse anti-transferrin receptor (13–6800, Invitrogen), or rabbit polyclonal anti-ferroportin 1 (NBP1-21502SS, Novus Biologicals). Membranes were washed and incubated with secondary antibodies for 1 hr at room temperature. Protein bands were visualized by chemiluminescence (PIERCE or Amersham) followed by exposure to autoradiographic film. For loading control, membranes were stripped in buffer containing 62.5mM Tris pH6.8, 2% SDS, and 100mM β-mercaptoethanol for 30 min at 55°C, washed extensively in running water, blocked in 5% non-fat milk for 1 hr at room temperature and incubated overnight at 4°C with mouse monoclonal antibody against β-tubulin (MAB 3408 Chemicon) or anti-actin (A4700 Sigma).

For quantification, membranes were scanned and band intensities were analyzed using the ImageJ software (http://rsb.info.nih.gov/ij/). The final relative quantification values were determined using the ratio of net band to net loading control.

### MSD electrochemiluminescence assays

Htt protein expression levels were independently analyzed by the biopharmaceutical company BioFocus, using MSD electrochemiluminescence CHDI_HTT_003 assay, as described [74]. Briefly, dissected cortex and striatum from 3 month-old and 6 month-old mice (n = 3 per sex, timepoint, and genotype) and 4 month-old and 7 month-old cKO mice TM-treated at 3 months of age and 6 months of age, respectively, were homogenized and analyzed as described [62]. The samples were tested in technical triplicates on separate MSD plates. Standard curves of purified N-terminal mouse Htt Q7 protein standard (NF549mQ7) were used to quantify mouse Htt protein. Assay variation between replicate plates averaged about 15% for all analyzed cortical samples and about 5% for striatal samples.

### SHIRPA

We used a modified version of the **S**mithKline Beecham, **H**arwell, **I**mperial College, **R**oyal London Hospital **P**henotype **A**ssessment (**SHIRPA**) protocol and we followed the protocol outlined in Glynn et al., 2003, which takes into account not only the number of abnormal traits but also scores their degree of severity. Mice were analyzed longitudinally. A normal behavior received a score of “0”. Higher scores indicate progression of severity of the abnormalities. Data were quantified using the modified SHIRPA scoring system outlined in the Table 1.

**Table 1:** Modified SHIRPA scoring system.

Test	Score = 0	Score = 1	Score = 2	Score = 3
Piloerection	None	Coat stands on end		
Domed face	Absent	Mild	Severe	
Palpebral closure	Eyes wide open	Eyes half closed	Eyes closed	
Eye fur	Normal	Slightly pale	Very pale	
Tail elevation	Horizontal	Elevated		
Lordokyphosis	Absent	Mild	Severe	
Hindlimb clasping	Absent	Tendency	Clenching on midline	Clasping immediately
Forelimb clasping	Absent	Tendency	Clasping immediately	
Tremor	None	Mild	Marked	
Gait	Normal	Abnormal	Limited movement	
Vocalization	None	Provoked by handling		
Initial activity	Alert, active	quiet	asleep	
Respiration rate	Normal	Gasping irregular	Shallow	

### Rotarod test

For rotarod tests we used a San Diego Instruments accelerating rotarod (San Diego, CA) using established protocols with minor modifications [75]. Mice were given 3 days to become acquainted with the rotarod apparatus before actual measurements were initiated. The average of length of time before the mouse falls off the rotating rod (latency to fall) was used as the measure of competency at this task.

### Histological analyses

For histology, brains and organs were collected and fixed in 4% paraformaldehyde in phosphate-buffered saline (PBS) for at least 1 week; incubated for 24 hours at 4°C in PBS containing 0.25 M sucrose, 0.2 M glycine; dehydrated; cleared with toluene; and embedded in paraffin. Paraffin blocks were sectioned at 7 μm, mounted in superfrost slides (Fisher) and stained with haematoxylin and eosin (H&E).

### Immunohistochemistry

For immunohistochemistry on paraffin sections, slides were deparaffinized, rehydrated, and incubated with 0.3% H_2_0_2_ in methanol for 20 min, to quench endogenous peroxidase. Sections were then washed with PBS, blocked for 1 hr with 4% BSA, 0.2% Triton X-100 in PBS, and incubated at 4°C for 48–72 hrs with primary rabbit polyclonal anti-DARPP-32 (AB 1656, Chemicon); mouse anti-Glial Fibrillary Protein (NE 1015, Calbiochem), rabbit monoclonal anti-calbindin (Catalog # 2946–1, Epitomics), or rabbit polyclonal anti-ferritin light chain (AB 69090, Abcam), in 0.4% BSA; 0.2% Triton X-100 in PBS. For detection of Glial Fibrillary Protein (GFAP), sections were first treated with 0.1% trypsin for 30 min at 37°C for antigen retrieval prior to blocking. No antigen retrieval was necessary for any of the other antibodies used. In all cases, following primary antibody incubation, slides were washed five times with PBS, and primary antibody detection was carried out using the Vector ABC kit according to manufacturers’ instructions, followed by incubation with DAB brown substrate (BD Biosciences).

Quantification of GFAP positive cells was performed as described [76]. Briefly, for each mouse, two images (one from each hemisphere) from two independently-stained coronal brain sections spanning the striatum were captured at 40X, divided into quadrants and a grid stereologic method was used to count the total number of GFAP-stained glial cell bodies in each quadrant using ImageJ. Student's *t*-test was used for comparisons between genotypes.

### Quantification of medium spiny neurons

Medium spiny neurons were quantified in 18mo CTL TM@6mo (n = 3) and 18mo cKO TM@6mo (n = 3) mice, by counting the numbers of DARPP-32 and calbindin- stained neuronal cell bodies in cross sections of the striatum. For each mouse, four images per striatal area of both hemispheres from two independently-stained forebrain coronal sections were captured at 10X and divided into quadrants. The number of cells per quadrant was counted using ImageJ.

### Quantification of calbindin-positive neurons in cortex layers 2 and 3

Calbindin-positive neurons in the cortex were quantified in 18mo CTL TM@6mo (n = 3) and 18mo cKO TM@6mo (n = 3) mice, by counting the numbers of calbindin- stained neuronal cell bodies in layers 2 and 3 of the cortex at bregma 1.10. For each mouse, two images (one from each hemisphere) from two independently-stained coronal brain sections were captured at 10X and divided into quadrants. The number of cells per quadrant was counted using ImageJ.

### Quantification of cortex thickness

Cortical thickness was measured on coronal brain sections of 18mo CTL TM@6mo (n = 6) and 18mo cKO TM@6mo (n = 6) mice. Briefly, for each mouse, two images from H&E-stained coronal brain sections around bregma 1.10 were captured at 10X and cortex thickness was measured on both hemispheres. Cortical thickness is defined as the distance, in microns, between the white matter and the layer I border.

### Histochemical staining for calcium

Sections were deparaffinized, hydrated to 70% ethanol, and briefly rinsed in distilled water. Slides were then incubated in 2% Alizarin Red S (Sigma A-5533) pH 4.3 (adjusted with ammonium hydroxide), and blotted to remove excess dye. Slides were then dipped 20 times in acetone, followed by 20 times in acetone-Citrisolv. Slides were then cleared in Citrisolv and mounted.

### Histochemical staining for iron

The histochemical distribution of Fe^3+^ was determined by Perls’ staining as described [36], with some modifications. For ferric iron detection in the calcified brain lesions, paraffin-embedded sections were deparaffinized, rehydrated, and incubated in 2% potassium ferrocyanide; 2% HCl solution for 45 minutes at room temperature. Slides were then rinsed in water and incubated with 0.3% H_2_0_2_ in methanol for 20 min, to quench endogenous peroxidase. Sections were then rinsed in water and incubated for 5 minutes in diamenobenzidine (DAB) with metal enhancer (Sigma FAST-DAB) followed by an additional 5 minutes in DAB in the presence of 0.3% H_2_0_2_. Slides were rinsed in water and counterstained with eosin. Incubation with DAB was omitted for iron detection in spleen sections, due to the high level of iron content in the spleen.

For tissues where iron content is normally low (brain and liver), an alternative protocol that enhances sensitivity was employed [77]. Briefly, slides were first deparaffinized with SafeClear (Fisher Scientific) and air-dried for 20 min at room temperature, followed by staining as described above. Quantification of Perls’ staining was performed as described [78]. Briefly, for each mouse two images from Perls’ stained coronal brain sections encompassing the cortex or the striatum were captured at 20X and divided into quadrants. Mean pixel intensity per quadrant was measured using ImageJ. Student's *t*-test was used for comparisons between genotypes.

In all cases, control sections were processed in parallel, in the absence of potassium ferrocyanide.

### Statistics

Data presented are shown as mean ± SD or SEM as specified under each figure. Data were derived from multiple independent experiments from distinct mice. No randomization of animals was performed, but animals were sex- and age-matched, and littermates were used whenever possible. Animal studies were performed without blinding of the investigator. Histological analyses were performed in at least 3 mice per time-point and genotype. No statistical method was used to predetermine sample size, but sample size was based on preliminary data and previous publications. In all experiments the differences were considered significant when P<0.05. Open access software was used in most cases. The differences between groups were assessed using one-way or two-way ANOVA followed by Bonferroni’s post hoc test. Student’s *t*-test was used for comparison of two groups. Analysis of survival by log-rank test was also performed.

## Supporting information

S1 Fig Evaluation and quantification of a tamoxifen inducible system for global Htt ablation. (A) Quantification of Htt expression in cKO mice at different ages. Western blots of total brain protein extracts from *Hdh*^*flox/-*^ and cKO noTM mice at 12, 15 and 22 months of age (n = 3 for each genotype and age) were probed with mouse monoclonal anti-htt 2166 antibody, stripped and re-probed with anti-β-tubulin antibody. Bands intensities were quantified using image J, and Htt levels were normalized over β-tubulin levels. Values represent the means of Htt expression in cKO brains over the means of Htt expression in the respective age-matched *Hdh*^*flox/-*^ brains. (*P<0.05, Student’s t-test). (B) Semi-quantitative RT-PCR analyses on total RNA from brains of 4 month-old *Hdh*^*flox/+*^ (flox/+), *Hdh*^*flox/-*^ (flox/-), untreated cKO and cKO TM-treated at 3 months of age (n = 5 for each genotype and condition) using primers specific for Hdh coding regions spanning exons 11 and 12. 18S rRNA amplification was used as internal control. Data are expressed as mean ± SD. Results are presented as percentage Hdh mRNA levels relative to *Hdh*^*flox/+*^ levels. One-way analysis of variance (ANOVA) followed by Bonferroni post hoc test. ***P<0.001 versus flox/+, ^#^P<0.001 versus flox/- and untreated cKO. (C) Western blots of total protein extracts from brains of 4mo cKO TM@3mo mice (cKO, n = 5) and controls (CTL, n = 5) were probed with mouse monoclonal anti-htt 2166 antibody (Chemicon), stripped and re-probed with anti-β-tubulin antibody. Bands intensities were quantitated using Image J. Htt levels were normalized over β-tubulin levels. Values represent mean relative to controls ± SD (***P<0.001, Student’s t-test). (D) Quantification of TM-induced Cre-mediated recombination at the DNA level. Total genomic DNA from brain and peripheral tissues from 6mo cKO TM@3mo mice (n = 4) was submitted to PCR using primers that amplify the unrecombined flox allele (unrec) and recombined Δflox Hdh allele (rec). Relative intensities were determined using imaging system F pro. Individual organ recombination is expressed as percent of total Hdh alleles (recombined and unrecombined). Data are represented as mean ± SD. (***P<0.001, Student’s t-test, unrec versus rec).
(TIF)

S2 Fig Schematic representation of the experimental design. Control (*Hdh*^*flox/+*^ and *Hdh*^*flox/-*^) and experimental CreER; *Hdh*^*flox/-*^ (cKO) mice were grouped into four cohorts: cohort #1 (untreated = no TM administration, only vehicle was injected), cohort #2 (TM administration at 3 months of age), cohort #3 (TM administration at 6 months of age), and cohort #4 (TM administration at 9 months of age). Female and male mice from all cohorts were monitored longitudinally for weight gain, SHIRPA and survival, starting at 2 months of age. Male and female mice from all cohorts were sacrificed at selected time-points for tissue collection and analyses.
(TIF)

S3 Fig Cumulative rotarod analyses of different cohorts of mice. Blue diamond CTL noTM (n = 15), green diamond CTL TM inj (n = 15), red diamond cKO noTM (n = 10), brown diamond cKO TM@9mo (n = 12), pink diamond cKO TM@6mo (n = 10), and black diamond cKO TM@3mo (n = 6). Data are represented as mean without error bars, so the dynamics of the curves are not obscured. Note that Htt elimination results to a steep reduction of rotarod performance.
(TIF)

S4 Fig Cumulative weight gain curves. Body weights of (A) males (blue diamond CTL noTM, n = 16; green diamond CTL TM inj, n = 10; red diamond cKO noTM, n = 10; pink diamond cKO TM@6mo, n = 9) and (B) females (blue diamond CTL noTM, n = 20; green diamond CTL TM inj, n = 15; red diamond cKO noTM, n = 10; pink diamond cKO TM@6mo, n = 8). Data are represented as mean without error bars, so the dynamics of weight gain are not obscured. Note that Htt elimination affects weight gain.
(TIF)

S5 Fig Eye abnormalities in TM-treated CreER; *Hdh*^*flox/-*^ mice. (A) lateral view of a normal eye from a 11mo CTL noTM and 11mo cKO TM@6mo. Note the thickened opaque cornea of the TM-treated cKO mouse eye. (B) H&E-stained cross-sections through the eyes of 17mo CTL TM@6mo, and 15mo cKO TM@9mo. Note the thickened keratinized cornea epithelium in the TM-treated cKO mouse eye section.
(TIF)

S6 Fig Rectal prolapse and testicular atrophy in mice lacking Htt. (A) 17mo CTL female mouse has normal anus, while 17mo cKO TM@6mo female mouse display severe rectal prolapse. Note the protrusion of the rectal mucosa. (B) Representative testes from 13mo CTL (left) and 13mo cKO TM@6mo (right).
(TIF)

S7 Fig Histological analyses of peripheral organs and tissues. Representative H&E-stained transverse sections of (A) kidney, (B) liver, (C) pancreas, (D) adipose tissue, (E) spleen, and (F) skeletal muscle of TM-treated CTL and cKO mice. Tissues were collected 9–14 months after TM administration. Note that peripheral tissues appear normal after long-term Htt elimination. G = glomerulus, IL = Islets of Langerhans, Ac = acini.
(TIF)

S8 Fig Quantification of GFAP-positive cells in brains of mice lacking Htt. (A) Density of GFAP-positive cells in the striatum of 18mo CTL TM@9mo (n = 4) and 18mo cKO TM@9mo (n = 4). (B) Density of GFAP-positive cells in the thalamus of 12mo CTL TM@9mo (n = 4) and 12mo cKO TM@9mo (n = 4). The number of GFAP-positive cells in the striatum and thalamus was quantified using image J as described in Materials and Methods. Values represent mean number of GFAP-positive cells per unit area. (***P<0.001, Student’s t-test).
(TIF)

S9 Fig Htt elimination does not result in calcium deposition in the kidney. Representative alizarin red S-stained cross sections of paraffin-embedded kidneys from 15mo CTL TM@3mo (A) and 15mo cKO TM@3mo (B). Note the absence of red-stained calcium deposits. G = glomerulus.
(TIF)

S10 Fig Quantification of Perls’ staining in the cortex of mice lacking Htt. Pixel intensity in the cortex of 18-19mo old CTL TM@9mo (n = 4) and 18-19mo old cKO TM@9mo (n = 4) was quantified using Image J. Values represent mean pixel intensity per unit area. (**P<0.01, Student’s t-test).
(TIF)

S11 Fig Htt elimination does not alter systemic heme-iron recycling. Representative Perls’ stained cross-sections of paraffin-embedded spleen and liver showing the localization of iron. (A) In the spleen, heme-iron recycling macrophages loaded with iron (blue staining) are equally distributed throughout the red pulp in 15mo CTL TM@3mo and 15mo cKO TM@3mo mice. (B) In the liver Perls’ stained (brown staining) iron-containing Kupffer cells (arrows) are present in both 15mo CTL TM@3mo and 15mo cKO TM@3mo mice.
(TIF)

S12 Fig Summary of major behavioral, histological and molecular observations. Red arrows indicate the time the described features were first observed.
(TIF)

S1 Table Weight gain of males noTM and TM-treated at 3 months. Male mice from different cohorts were weighted as described in Methods. Weight gain rate was calculated as dW/dt for each animal. Data are expressed as mean ± SD, and n = number of mice examined.
(DOCX)

S2 Table Weight gain of males noTM and TM-treated at 6 months. Male mice from different cohorts were weighted as described in Methods. Weight gain rate was calculated as dW/dt for each animal. Data are expressed as mean ± SD, and n = number of mice examined.
(DOCX)

S3 Table Weight gain of males noTM and TM-treated at 9 months. Male mice from different cohorts were weighted as described in Methods. Weight gain rate was calculated as dW/dt for each animal. Data are expressed as mean ± SD, and n = number of mice examined.
(DOCX)

S4 Table Weight gain of females noTM and TM-treated at 3 months. Female mice from different cohorts were weighted as described in Methods. Weight gain rate was calculated as dW/dt for each animal. Data are expressed as mean ± SD, and n = number of mice examined.
(DOCX)

S5 Table Weight gain of females noTM and TM-treated at 6 months. Female mice from different cohorts were weighted as described in Methods. Weight gain rate was calculated as dW/dt for each animal. Data are expressed as mean ± SD, and n = number of mice examined.
(DOCX)

S6 Table Weight gain of females noTM and TM-treated at 9 months. Female mice from different cohorts were weighted as described in Methods. Weight gain rate was calculated as dW/dt for each animal. Data are expressed as mean ± SD, and n = number of mice examined.
(DOCX)

S7 Table Testicular atrophy in mice lacking Htt. Testes from control (noTM and TM-treated at 9 months of age) and of cKO (noTM and TM-treated at 9 months of age) were collected from male mice ranging from 18 to 22 months of age, fixed in 4% PFA and weighed. For each animal examined, both testes were weighed and the mean was determined and used as absolute value for each mouse. Data are expressed as mean ± SD and n = number of mice examined. One-way analysis of variance (ANOVA) followed by Bonferroni post hoc test, ***P<0.001 versus CTL noTM, CTL TM@9mo and cKO noTM.
(DOCX)

S8 Table Organ weights. Organs from female and male mice from different cohorts were weighted as described. Data are represented as mean ± SD and n = number of mice examined. Except for brain and testes no significant differences were observed in other organs.
(DOCX)

S9 Table Htt elimination does not alter cortical thickness or calbindin neuronal numbers. Cortices from 18mo CTL TM@6mo and 18mo cKO TM@6mo mice were analyzed as described in Methods. Data are expressed as mean ± SD, and n = number of mice examined. No significant differences were observed.
(DOCX)

S10 Table Htt elimination does not alter striatal medium spiny neuronal numbers. Striata from 18mo CTL TM@6mo and 18mo cKO TM@6mo mice were analyzed as described in Methods. Data are expressed as mean ± SD, and n = number of mice examined. No significant differences were observed.
(DOCX)

S1 Movie 17 month-old male *Hdh*^*flox/+*^ injected with TM at 6 months of age. (MOV)

S2 Movie 17 month-old male CreER; *Hdh*^*flox/-*^ injected with TM at 6 months of age. Note the severe ataxic gait as the mouse walks. Gait disturbance in mice lacking htt is first noticeable about 6 months after Htt elimination, and progresses over time. In parallel, mice also develop hindlimb weakness, so that by end-stage they are unable to stand-up to feed and trail their hindlimbs as they walk around the cage.
(MOV)
