# Supplementary figures and images for: Elimination of huntingtin in the adult mouse leads to progressive behavioral deficits, bilateral thalamic calcification, and altered brain iron homeostasis
Source: PLoS Genet. 2017 Jul 17;13(7):e1006846. doi: 10.1371/journal.pgen.1006846 (PMC5536499; doi:10.1371/journal.pgen.1006846)

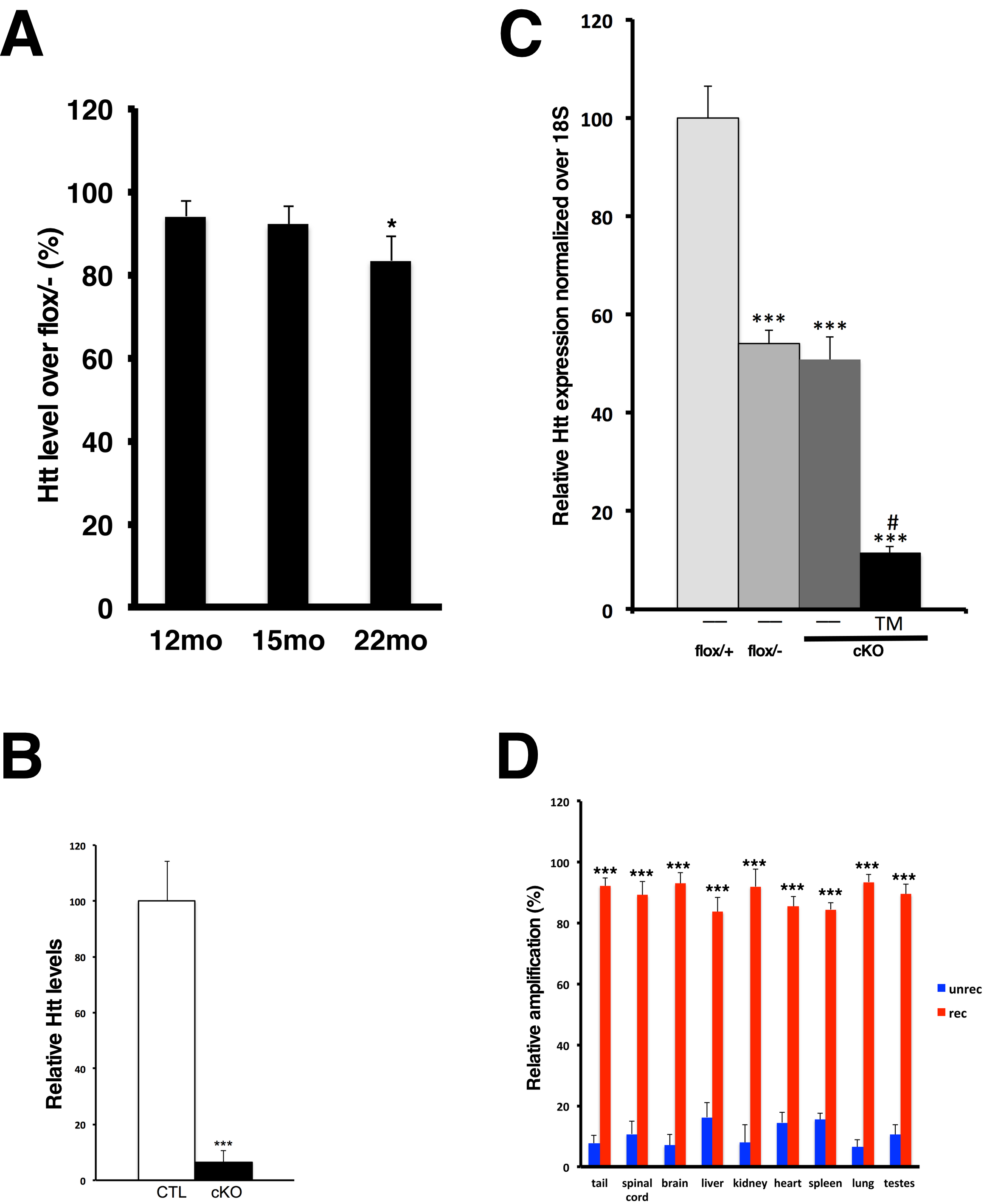

Supplement: S1 Fig — (A) Quantification of Htt expression in cKO mice at different ages. Western blots of total brain protein extracts from Hdhflox/- and cKO noTM mice at 12, 15 and 22 months of age (n = 3 for each genotype and age) were probed with mouse monoclonal anti-htt 2166 antibody, stripped and re-probed with anti-β-tubulin antibody. Bands intensities were quantified using image J, and Htt levels were normalized over β-tubulin levels. Values represent the means of Htt expression in cKO brains over the means of Htt expression in the respective age-matched Hdhflox/- brains. (*P<0.05, Student’s t-test). (B) Semi-quantitative RT-PCR analyses on total RNA from brains of 4 month-old Hdhflox/+ (flox/+), Hdhflox/- (flox/-), untreated cKO and cKO TM-treated at 3 months of age (n = 5 for each genotype and condition) using primers specific for Hdh coding regions spanning exons 11 and 12. 18S rRNA amplification was used as internal control. Data are expressed as mean ± SD. Results are presented as percentage Hdh mRNA levels relative to Hdhflox/+ levels. One-way analysis of variance (ANOVA) followed by Bonferroni post hoc test. ***P<0.001 versus flox/+, #P<0.001 versus flox/- and untreated cKO. (C) Western blots of total protein extracts from brains of 4mo cKO TM@3mo mice (cKO, n = 5) and controls (CTL, n = 5) were probed with mouse monoclonal anti-htt 2166 antibody (Chemicon), stripped and re-probed with anti-β-tubulin antibody. Bands intensities were quantitated using Image J. Htt levels were normalized over β-tubulin levels. Values represent mean relative to controls ± SD (***P<0.001, Student’s t-test). (D) Quantification of TM-induced Cre-mediated recombination at the DNA level. Total genomic DNA from brain and peripheral tissues from 6mo cKO TM@3mo mice (n = 4) was submitted to PCR using primers that amplify the unrecombined flox allele (unrec) and recombined Δflox Hdh allele (rec). Relative intensities were determined using imaging system F pro. Individual organ recombination is expres [file pgen.1006846.s001.tif]

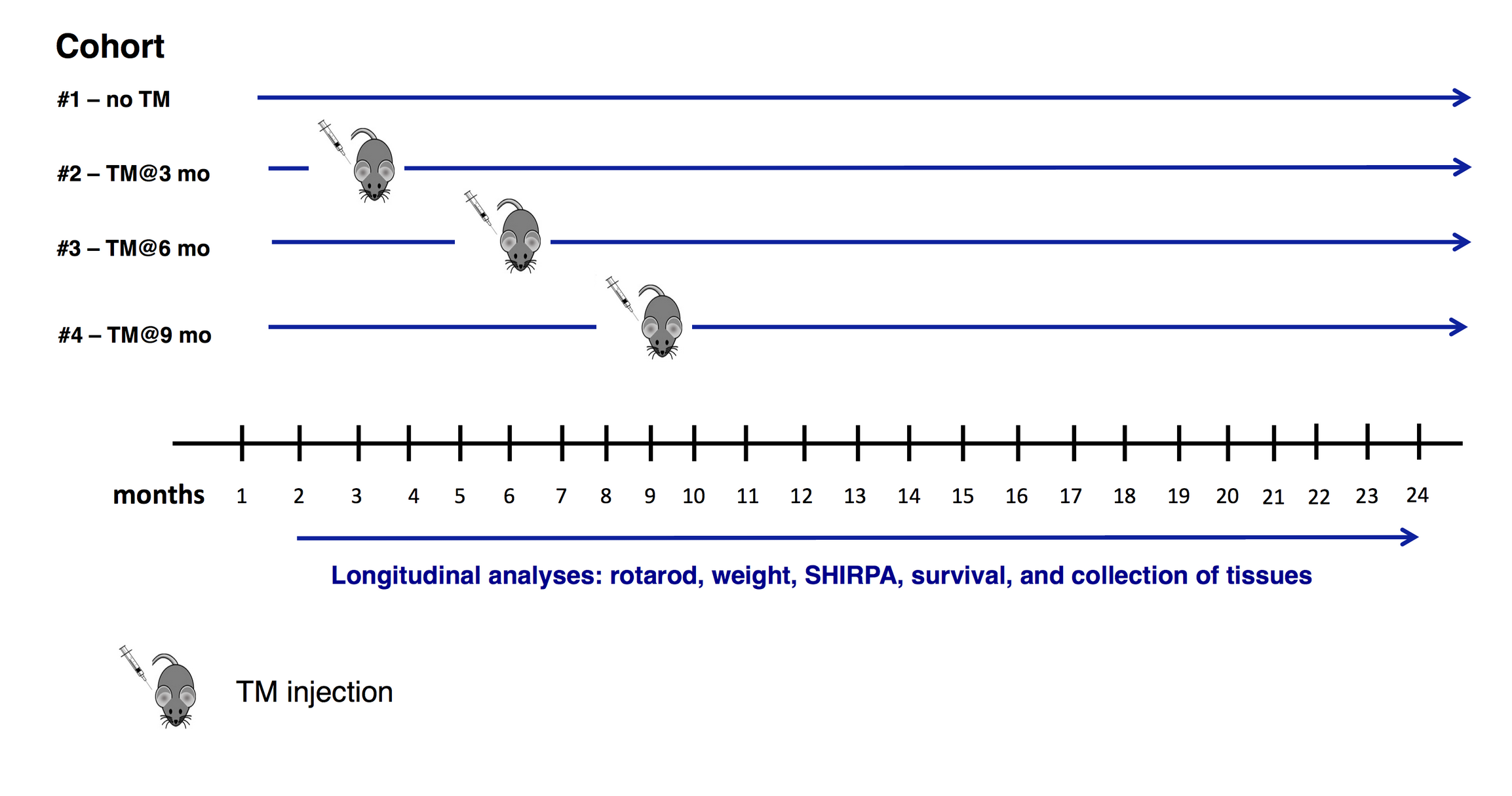

Supplement: S2 Fig — Control (Hdhflox/+ and Hdhflox/-) and experimental CreER; Hdhflox/- (cKO) mice were grouped into four cohorts: cohort #1 (untreated = no TM administration, only vehicle was injected), cohort #2 (TM administration at 3 months of age), cohort #3 (TM administration at 6 months of age), and cohort #4 (TM administration at 9 months of age). Female and male mice from all cohorts were monitored longitudinally for weight gain, SHIRPA and survival, starting at 2 months of age. Male and female mice from all cohorts were sacrificed at selected time-points for tissue collection and analyses. (TIF) [file pgen.1006846.s002.tif]

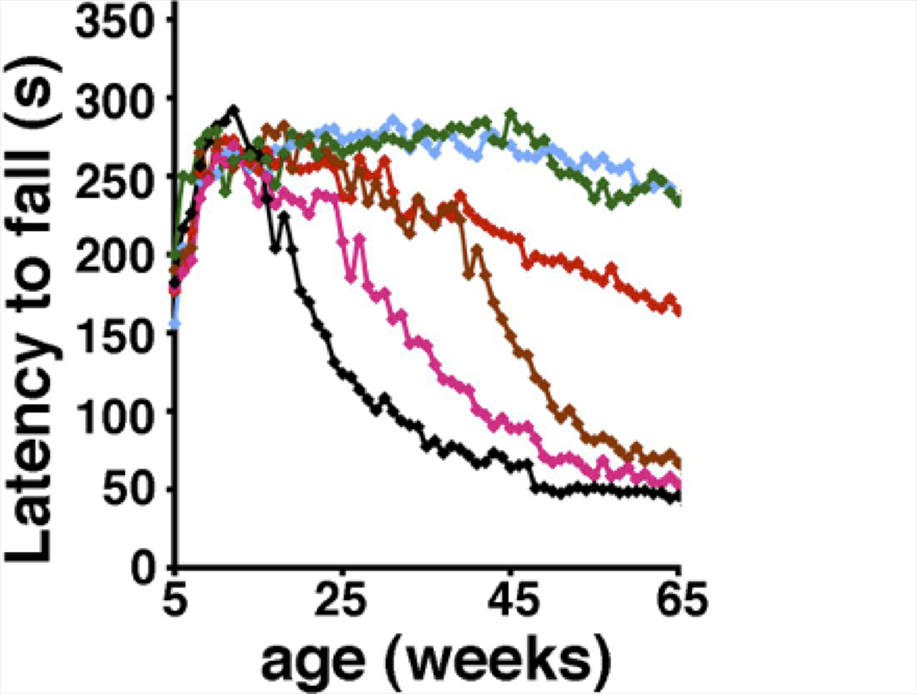

Supplement: S3 Fig — Blue diamond CTL noTM (n = 15), green diamond CTL TM inj (n = 15), red diamond cKO noTM (n = 10), brown diamond cKO TM@9mo (n = 12), pink diamond cKO TM@6mo (n = 10), and black diamond cKO TM@3mo (n = 6). Data are represented as mean without error bars, so the dynamics of the curves are not obscured. Note that Htt elimination results to a steep reduction of rotarod performance. (TIF) [file pgen.1006846.s003.tif]

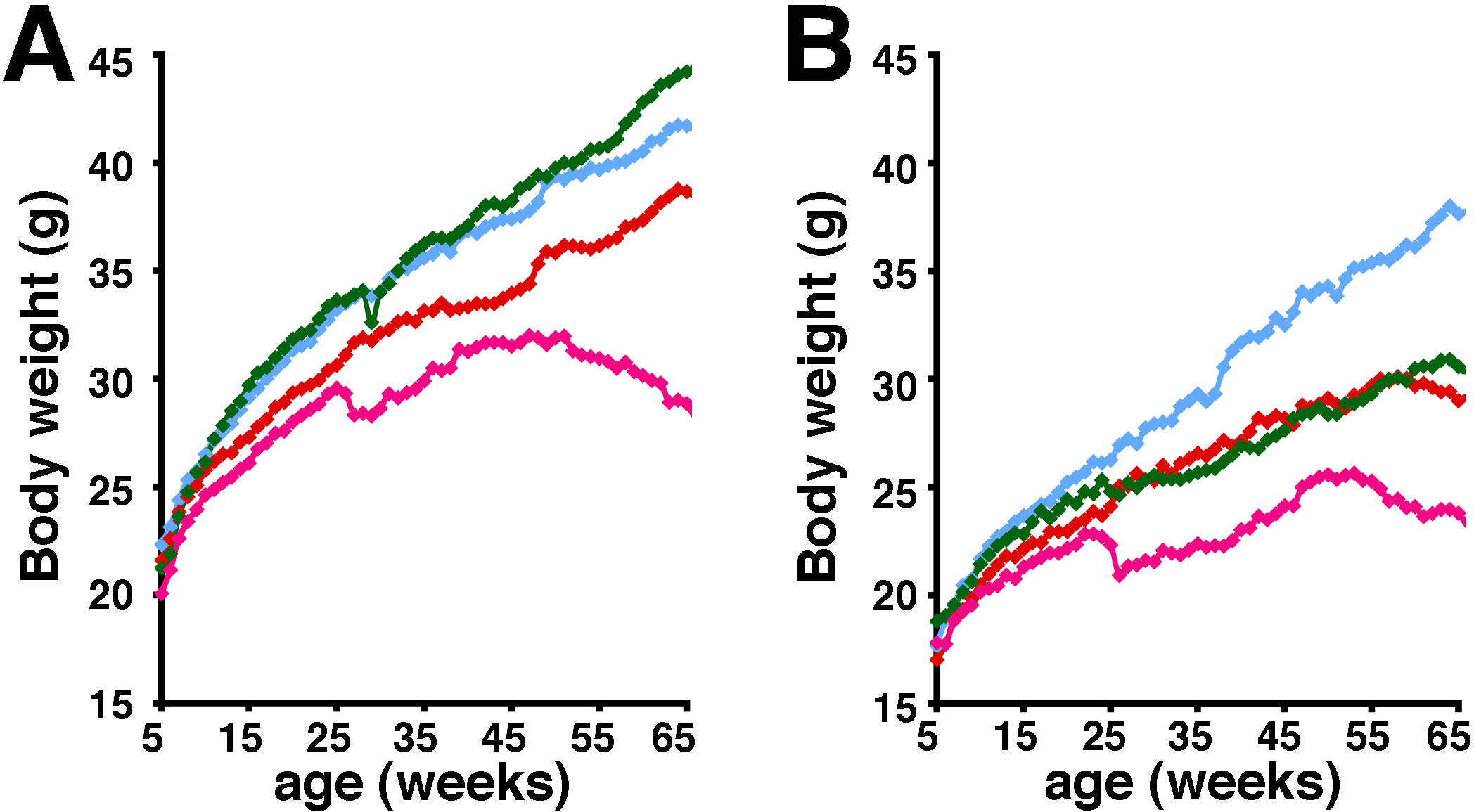

Supplement: S4 Fig — Body weights of (A) males (blue diamond CTL noTM, n = 16; green diamond CTL TM inj, n = 10; red diamond cKO noTM, n = 10; pink diamond cKO TM@6mo, n = 9) and (B) females (blue diamond CTL noTM, n = 20; green diamond CTL TM inj, n = 15; red diamond cKO noTM, n = 10; pink diamond cKO TM@6mo, n = 8). Data are represented as mean without error bars, so the dynamics of weight gain are not obscured. Note that Htt elimination affects weight gain. (TIF) [file pgen.1006846.s004.tif]

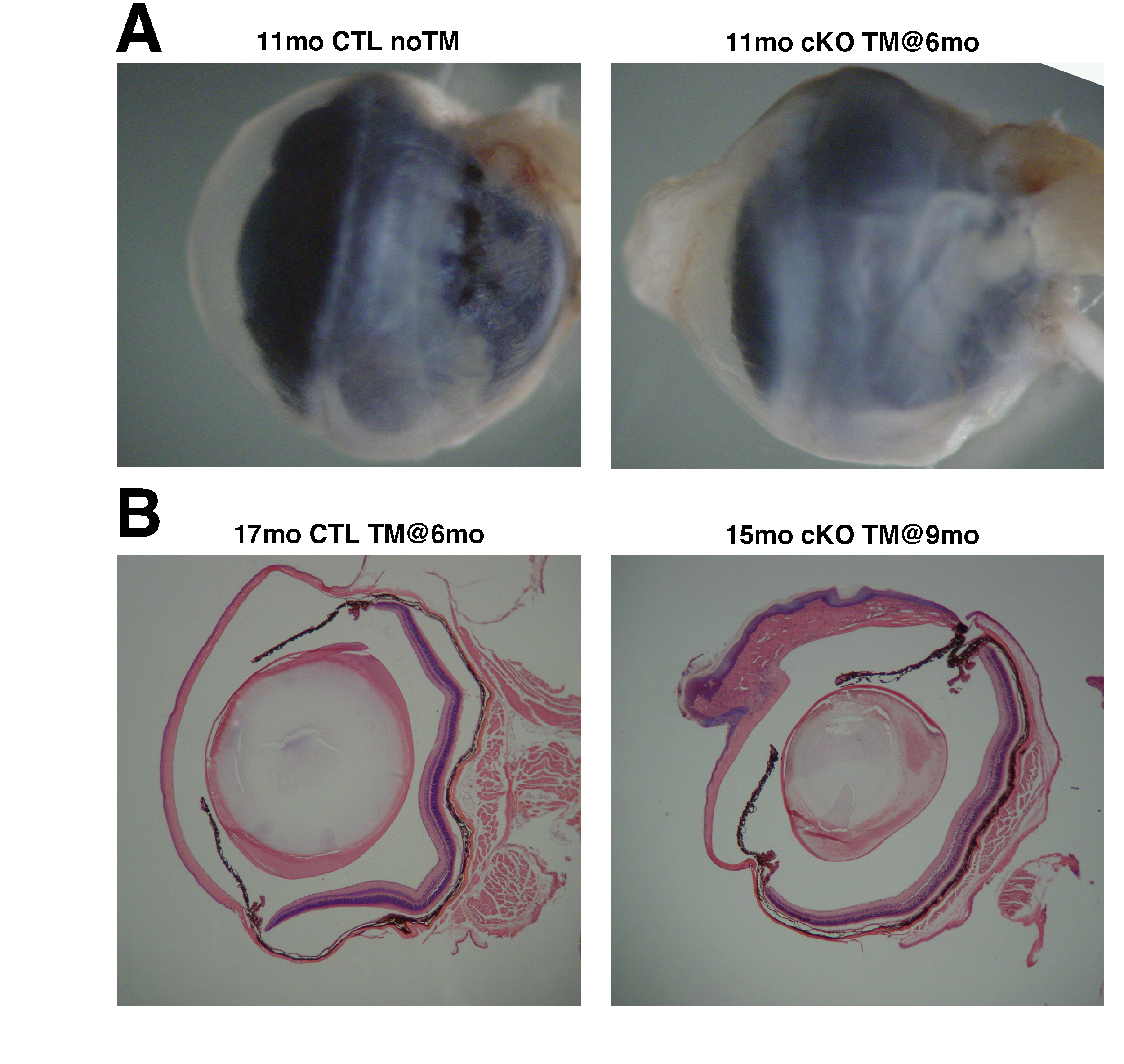

Supplement: S5 Fig — (A) lateral view of a normal eye from a 11mo CTL noTM and 11mo cKO TM@6mo. Note the thickened opaque cornea of the TM-treated cKO mouse eye. (B) H&E-stained cross-sections through the eyes of 17mo CTL TM@6mo, and 15mo cKO TM@9mo. Note the thickened keratinized cornea epithelium in the TM-treated cKO mouse eye section. (TIF) [file pgen.1006846.s005.tif]

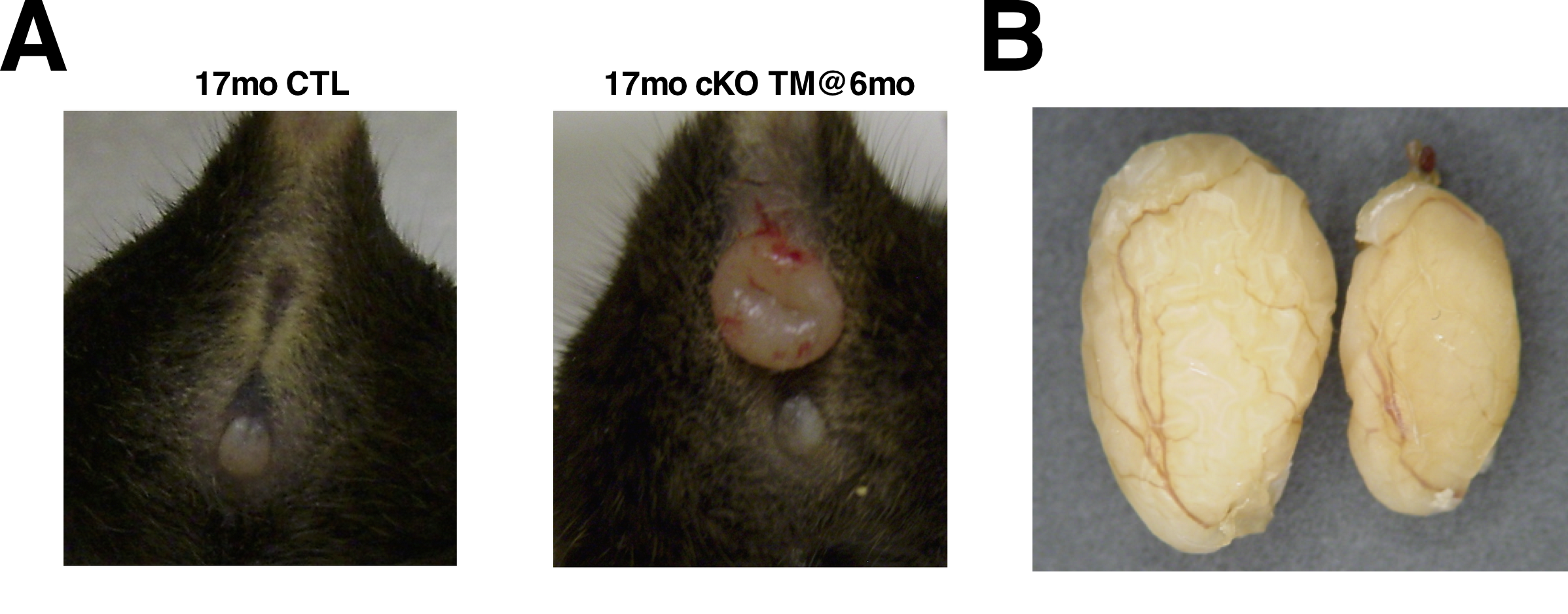

Supplement: S6 Fig — (A) 17mo CTL female mouse has normal anus, while 17mo cKO TM@6mo female mouse display severe rectal prolapse. Note the protrusion of the rectal mucosa. (B) Representative testes from 13mo CTL (left) and 13mo cKO TM@6mo (right). (TIF) [file pgen.1006846.s006.tif]

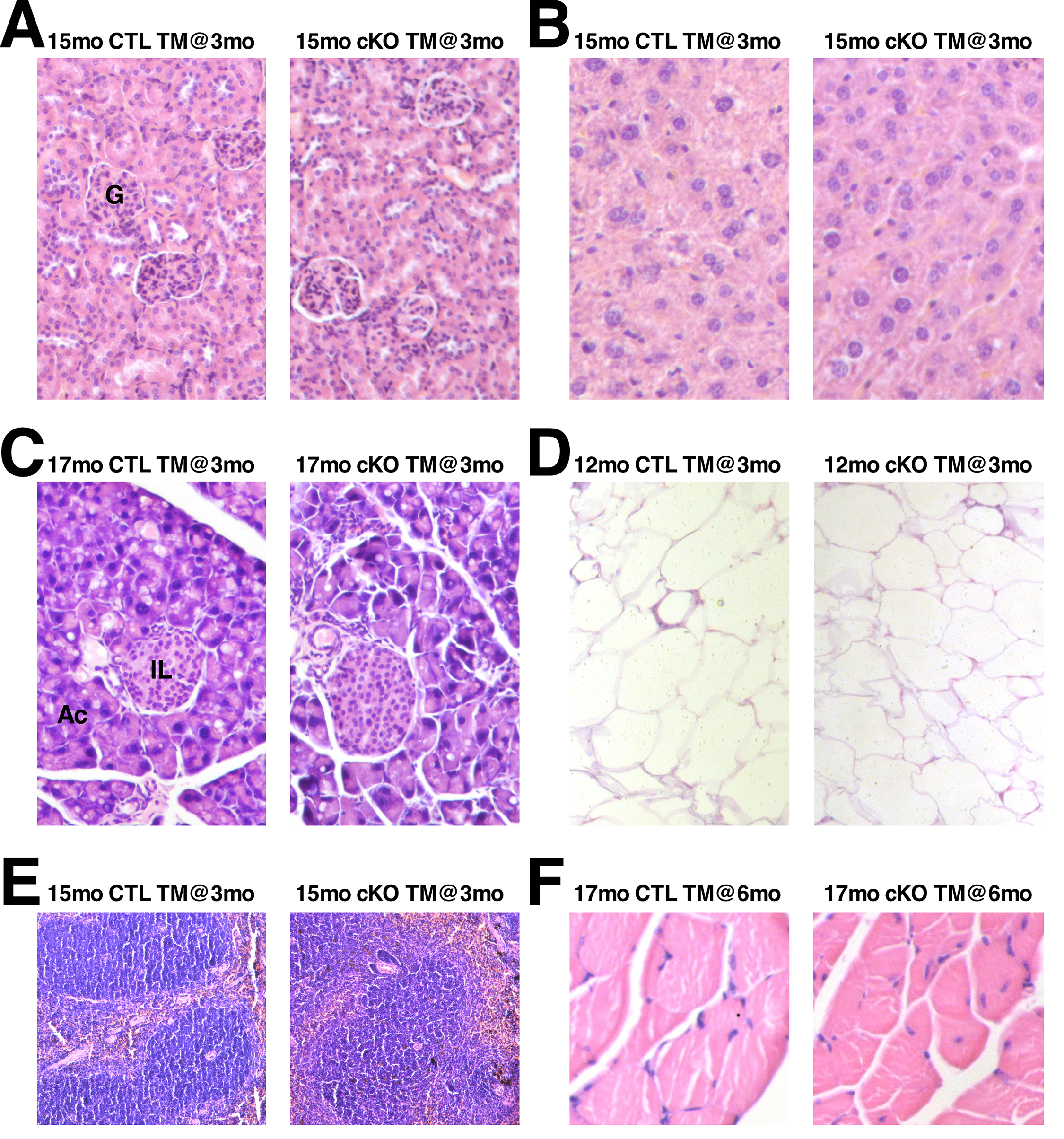

Supplement: S7 Fig — Representative H&E-stained transverse sections of (A) kidney, (B) liver, (C) pancreas, (D) adipose tissue, (E) spleen, and (F) skeletal muscle of TM-treated CTL and cKO mice. Tissues were collected 9–14 months after TM administration. Note that peripheral tissues appear normal after long-term Htt elimination. G = glomerulus, IL = Islets of Langerhans, Ac = acini. (TIF) [file pgen.1006846.s007.tif]

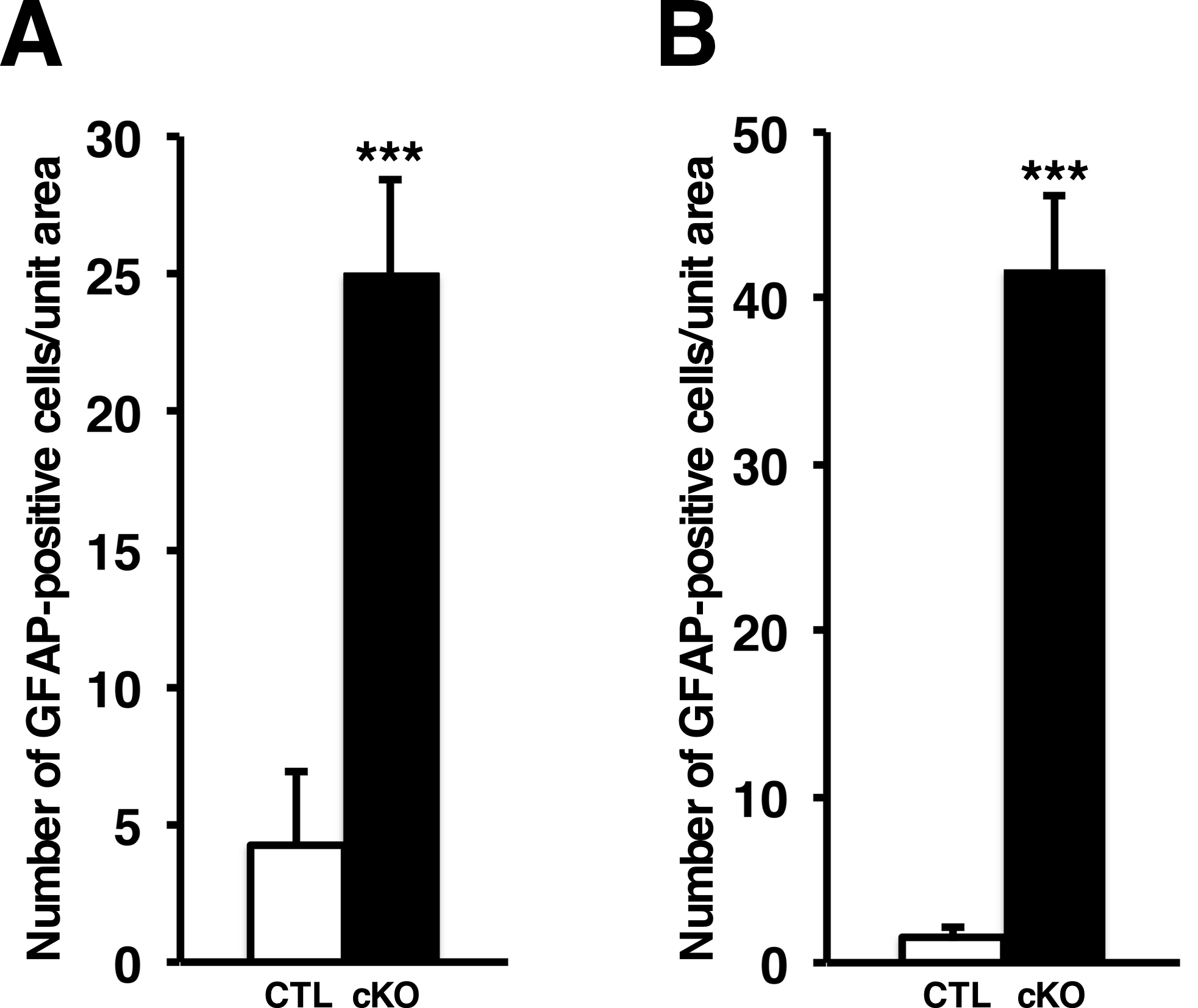

Supplement: S8 Fig — (A) Density of GFAP-positive cells in the striatum of 18mo CTL TM@9mo (n = 4) and 18mo cKO TM@9mo (n = 4). (B) Density of GFAP-positive cells in the thalamus of 12mo CTL TM@9mo (n = 4) and 12mo cKO TM@9mo (n = 4). The number of GFAP-positive cells in the striatum and thalamus was quantified using image J as described in Materials and Methods. Values represent mean number of GFAP-positive cells per unit area. (***P<0.001, Student’s t-test). (TIF) [file pgen.1006846.s008.tif]

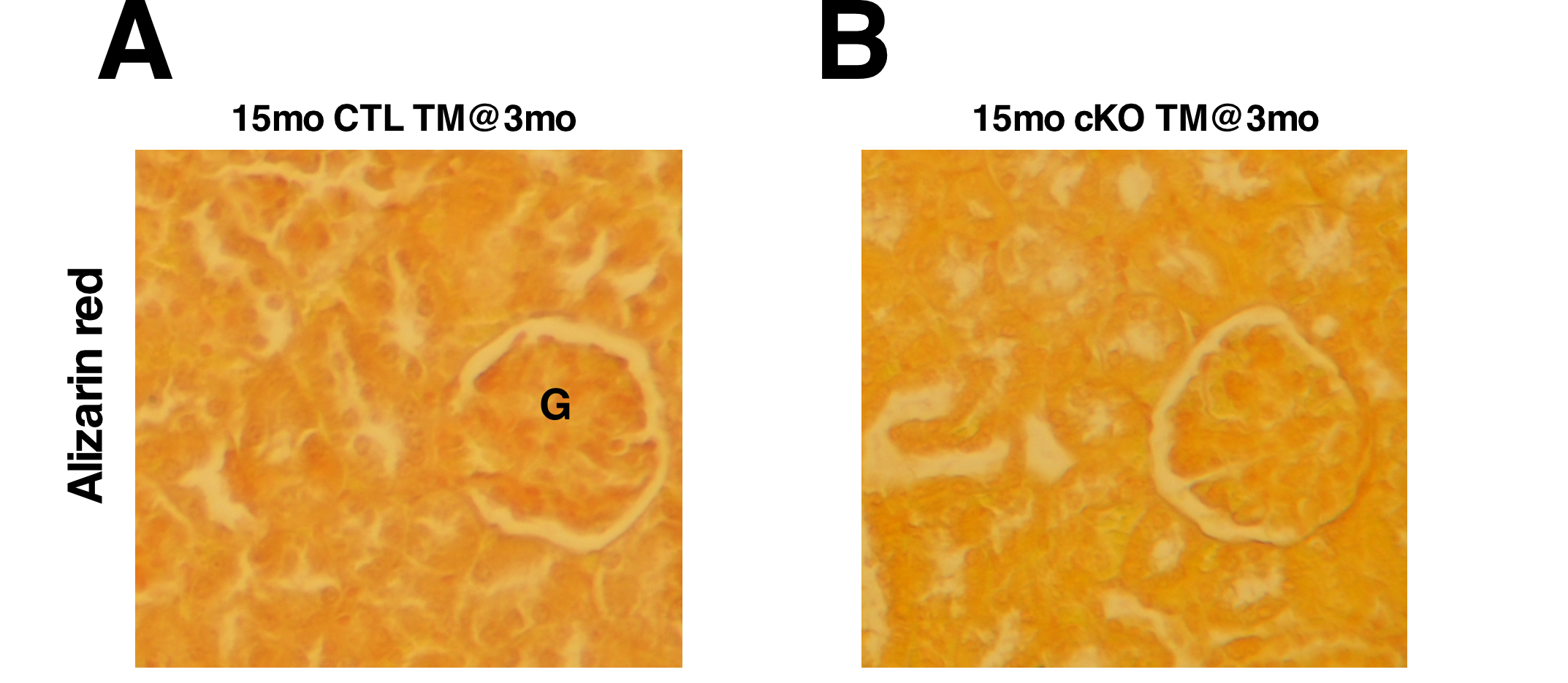

Supplement: S9 Fig — Representative alizarin red S-stained cross sections of paraffin-embedded kidneys from 15mo CTL TM@3mo (A) and 15mo cKO TM@3mo (B). Note the absence of red-stained calcium deposits. G = glomerulus. (TIF) [file pgen.1006846.s009.tif]

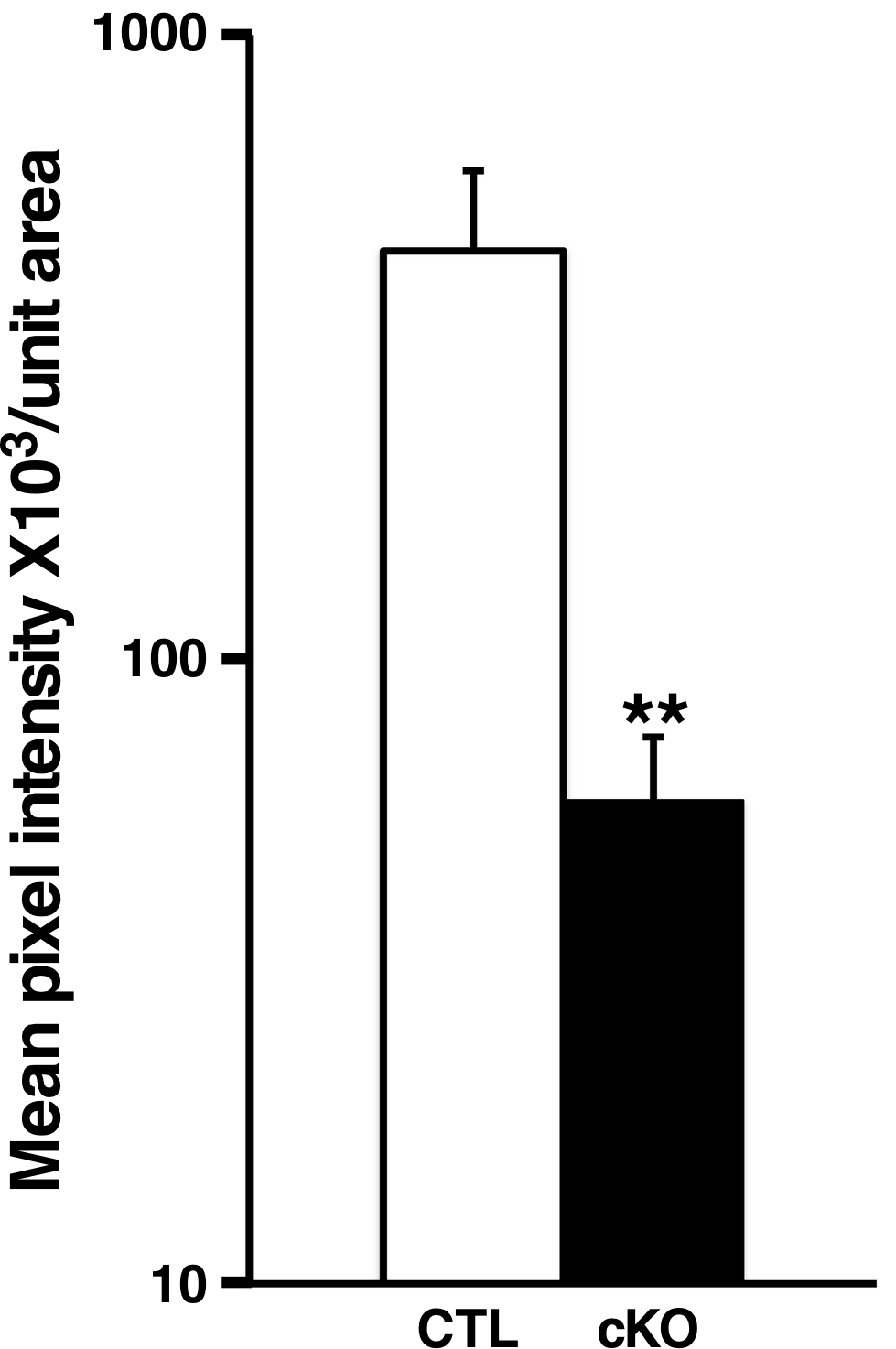

Supplement: S10 Fig — Pixel intensity in the cortex of 18-19mo old CTL TM@9mo (n = 4) and 18-19mo old cKO TM@9mo (n = 4) was quantified using Image J. Values represent mean pixel intensity per unit area. (**P<0.01, Student’s t-test). (TIF) [file pgen.1006846.s010.tif]

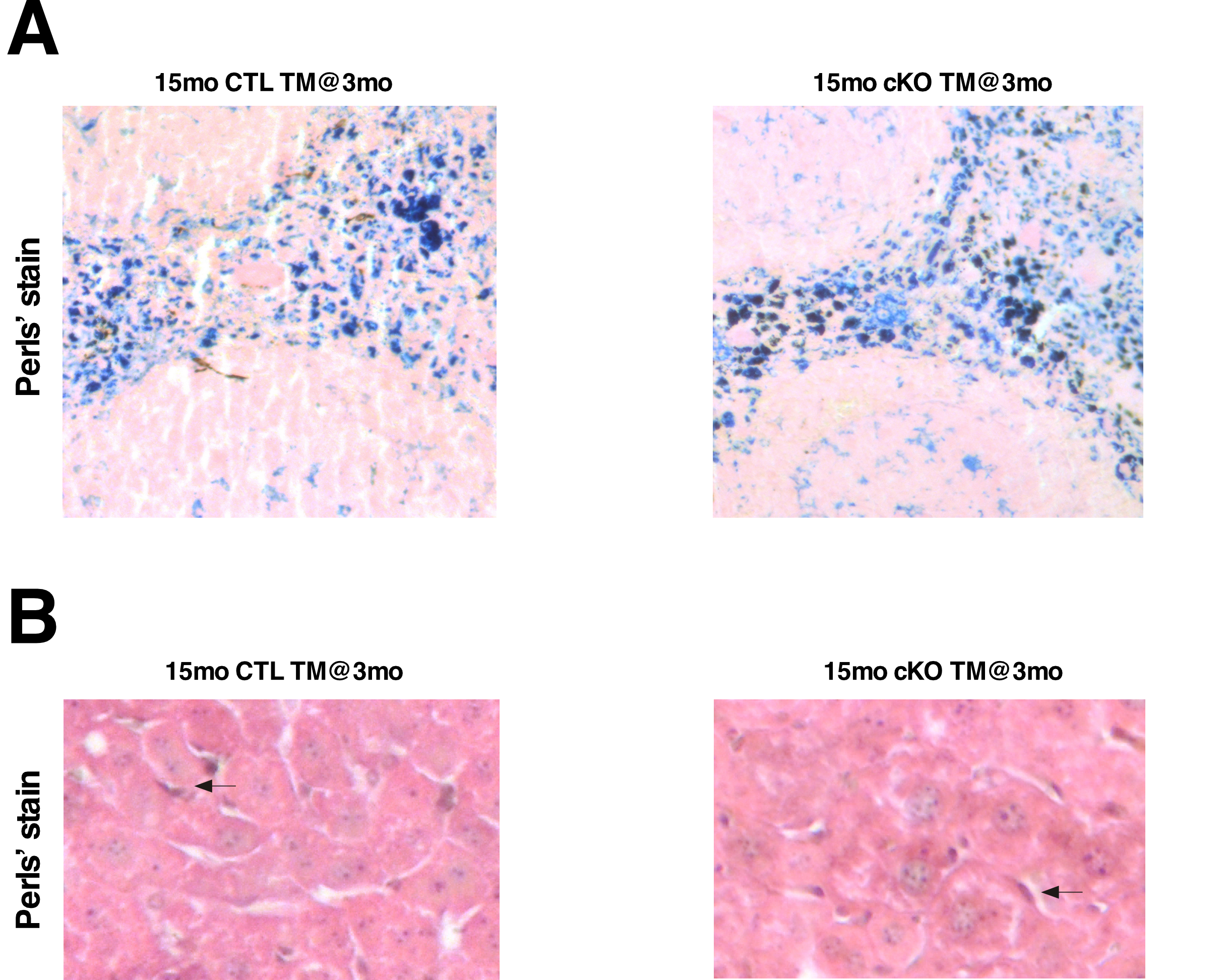

Supplement: S11 Fig — Representative Perls’ stained cross-sections of paraffin-embedded spleen and liver showing the localization of iron. (A) In the spleen, heme-iron recycling macrophages loaded with iron (blue staining) are equally distributed throughout the red pulp in 15mo CTL TM@3mo and 15mo cKO TM@3mo mice. (B) In the liver Perls’ stained (brown staining) iron-containing Kupffer cells (arrows) are present in both 15mo CTL TM@3mo and 15mo cKO TM@3mo mice. (TIF) [file pgen.1006846.s011.tif]

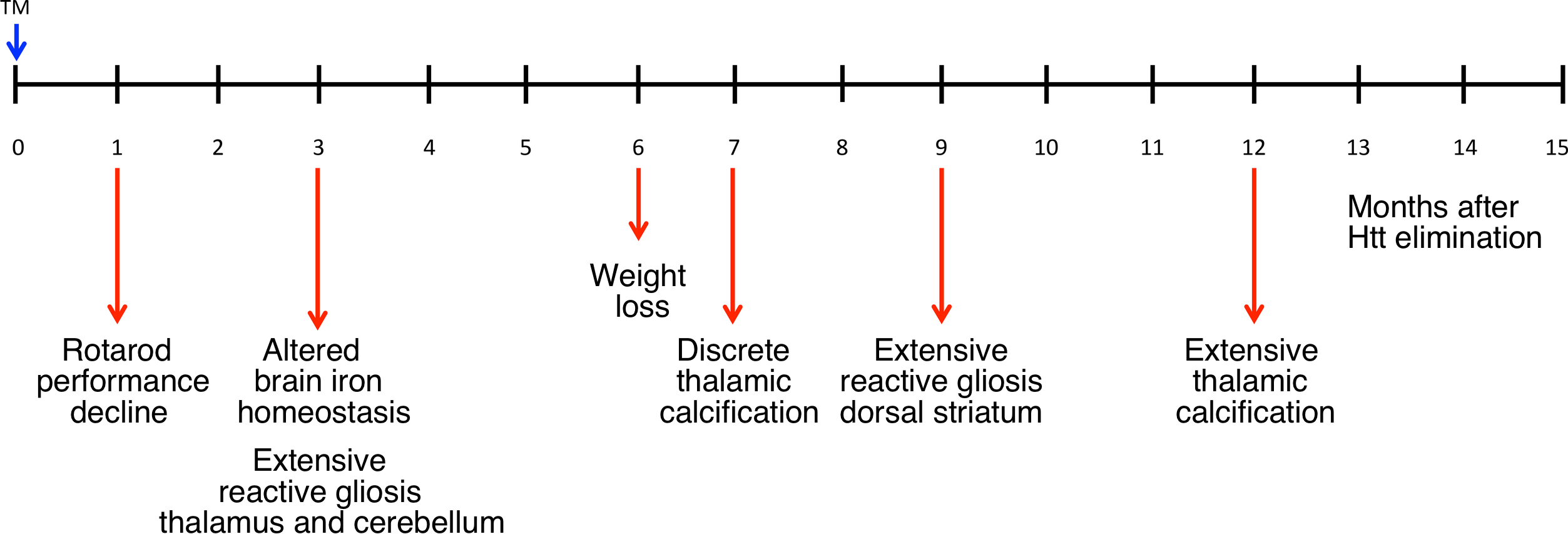

Supplement: S12 Fig — Red arrows indicate the time the described features were first observed. (TIF) [file pgen.1006846.s012.tif]
