# Supplementary material for: Elimination of huntingtin in the adult mouse leads to progressive behavioral deficits, bilateral thalamic calcification, and altered brain iron homeostasis
Source: PLoS Genet. 2017 Jul 17;13(7):e1006846. doi: 10.1371/journal.pgen.1006846 (PMC5536499; doi:10.1371/journal.pgen.1006846)
Supplement: S1 Table — Male mice from different cohorts were weighted as described in Methods. Weight gain rate was calculated as dW/dt for each animal. Data are expressed as mean ± SD, and n = number of mice examined. (DOCX) [file pgen.1006846.s013.docx]

**S1 Table. Male mice: weight data (12 – 65 weeks)**

| Genotype (number of mice) | 12 weeks | 65 weeks | Weight gain rate |
| --- | --- | --- | --- |
| CTL noTM (n=15) | 27.85±2.91 | 41.71±6.02 | 0.261±0.095 |
| CTL TM@3mo (n=7) | 27.86±2.71 | 39.66±4.28 | 0.223±0.080 |
| cKO noTM (n=8) | 26.14±2.18 | 39.03±5.39 | 0.243±0.071 |
| cKO TM@3mo (n=7) | 27.46±1.85 | 34.77±2.39 | 0.138±0.040^*^ |

Differences between groups were determined by one-way analysis of variance (ANOVA) followed by Bonferroni post hoc test. ^*^P<0.05 versus CTL noTM.
