# Supplementary material for: Elimination of huntingtin in the adult mouse leads to progressive behavioral deficits, bilateral thalamic calcification, and altered brain iron homeostasis
Source: PLoS Genet. 2017 Jul 17;13(7):e1006846. doi: 10.1371/journal.pgen.1006846 (PMC5536499; doi:10.1371/journal.pgen.1006846)
Supplement: S2 Table — Male mice from different cohorts were weighted as described in Methods. Weight gain rate was calculated as dW/dt for each animal. Data are expressed as mean ± SD, and n = number of mice examined. (DOCX) [file pgen.1006846.s014.docx]

**S2 Table. Male mice: weight data (24 – 65 weeks)**

| Genotype (number of mice) | 24 weeks | 65 weeks | Weight gain rate |
| --- | --- | --- | --- |
| CTL noTM (n=15) | 33.41±3.53 | 41.71±6.02 | 0.202±0.092 |
| CTL TM@6mo (n=8) | 33.74±5.07 | 43.94±5.01 | 0.249±0.042 |
| cKO noTM (n=8) | 30.56±3.52 | 39.03±5.39 | 0.206±0.064 |
| cKO TM@6mo (n=7) | 29.44±1.76 | 28.94±2.03 | -0.012±0.028*** |

Differences between groups were determined by one-way analysis of variance (ANOVA) followed by Bonferroni post hoc test. ***P<0.001 versus CTL noTM, CTL TM@6mo and cKO noTM.
