# Supplementary material for: Elimination of huntingtin in the adult mouse leads to progressive behavioral deficits, bilateral thalamic calcification, and altered brain iron homeostasis
Source: PLoS Genet. 2017 Jul 17;13(7):e1006846. doi: 10.1371/journal.pgen.1006846 (PMC5536499; doi:10.1371/journal.pgen.1006846)
Supplement: S3 Table — Male mice from different cohorts were weighted as described in Methods. Weight gain rate was calculated as dW/dt for each animal. Data are expressed as mean ± SD, and n = number of mice examined. (DOCX) [file pgen.1006846.s015.docx]

**S3 Table. Male mice: weight data (36 – 65 weeks)**

| Genotype (number of mice) | 36 weeks | 65 weeks | Weight gain rate |
| --- | --- | --- | --- |
| CTL noTM (n=15) | 37.22±4.30 | 41.71±6.02 | 0.155±0.130 |
| CTL TM@9mo (n=9) | 33.88±3.39 | 39.17±4.19 | 0.182±0.051 |
| cKO noTM (n=8) | 33.35±4.27 | 39.03±5.39 | 0.196±0.062 |
| cKO TM@9mo (n=8) | 33.25±2.52 | 33.19±2.07 | -0.005±0.103** |

Differences between groups were determined by one-way analysis of variance (ANOVA) followed by Bonferroni post hoc test. **P<0.01 versus CTL noTM, CTL TM@9mo and cKO noTM.
