# Supplementary material for: Elimination of huntingtin in the adult mouse leads to progressive behavioral deficits, bilateral thalamic calcification, and altered brain iron homeostasis
Source: PLoS Genet. 2017 Jul 17;13(7):e1006846. doi: 10.1371/journal.pgen.1006846 (PMC5536499; doi:10.1371/journal.pgen.1006846)
Supplement: S4 Table — Female mice from different cohorts were weighted as described in Methods. Weight gain rate was calculated as dW/dt for each animal. Data are expressed as mean ± SD, and n = number of mice examined. (DOCX) [file pgen.1006846.s016.docx]

**S4 Table. Female mice: weight data (12 – 65 weeks)**

| Genotype (number of mice) | 12 weeks | 65 weeks | Weight gain rate |
| --- | --- | --- | --- |
| CTL noTM (n=18) | 23.02±3.16 | 37.41±5.03 | 0.272±0.063 |
| CTL TM@3mo (n=9) | 23.18±4.31 | 31.14±3.86 | 0.150±0.026^a^ |
| cKO noTM (n=8) | 21.75±2.72 | 30.21±5.23 | 0.160±0.066^a^ |
| cKO TM@3mo (n=7) | 22.84±1.99 | 25.20±1.67 | 0.044±0.014^a,b,c^ |

Differences between groups were determined by one-way analysis of variance (ANOVA) followed by Bonferroni post hoc test. ^a^P<0.001 versus CTL noTM, ^b^P<0.01 versus CTL TM@3mo and ^c^P<0.001 versus cKO noTM.
