# Supplementary material for: Elimination of huntingtin in the adult mouse leads to progressive behavioral deficits, bilateral thalamic calcification, and altered brain iron homeostasis
Source: PLoS Genet. 2017 Jul 17;13(7):e1006846. doi: 10.1371/journal.pgen.1006846 (PMC5536499; doi:10.1371/journal.pgen.1006846)
Supplement: S5 Table — Female mice from different cohorts were weighted as described in Methods. Weight gain rate was calculated as dW/dt for each animal. Data are expressed as mean ± SD, and n = number of mice examined. (DOCX) [file pgen.1006846.s017.docx]

**S5 Table. Female mice: weight data (24 – 65 weeks)**

| Genotype (number of mice) | 24 weeks | 65 weeks | Weight gain rate |
| --- | --- | --- | --- |
| CTL noTM (n=18) | 27.06±3.26 | 37.41±5.03 | 0.252±0.071 |
| CTL TM@6mo (n=9) | 25.33±3.51 | 31.08±5.15 | 0.140±0.047^a^ |
| cKO noTM (n=8) | 24.05±3.14 | 30.21±5.23 | 0.150±0.069^b^ |
| cKO TM@6mo (n=7) | 23.28±2.16 | 24.94±2.69 | 0.040±0.020^a,c^ |

Differences between groups were determined by one-way analysis of variance (ANOVA) followed by Bonferroni post hoc test. ^a^P<0.001 versus CTL noTM, ^b^P<0.01 versus CTL no TM, and ^c^P<0.01 versus CTL TM@6mo and cKO noTM.
