# Supplementary material for: Elimination of huntingtin in the adult mouse leads to progressive behavioral deficits, bilateral thalamic calcification, and altered brain iron homeostasis
Source: PLoS Genet. 2017 Jul 17;13(7):e1006846. doi: 10.1371/journal.pgen.1006846 (PMC5536499; doi:10.1371/journal.pgen.1006846)
Supplement: S6 Table — Female mice from different cohorts were weighted as described in Methods. Weight gain rate was calculated as dW/dt for each animal. Data are expressed as mean ± SD, and n = number of mice examined. (DOCX) [file pgen.1006846.s018.docx]

**S6 Table. Female mice: weight data (36 – 65 weeks)**

| Genotype (number of mice) | 36 weeks | 65 weeks | Weight gain rate |
| --- | --- | --- | --- |
| CTL noTM (n=18) | 30.95±4.14 | 37.41±5.03 | 0.223±0.066 |
| CTL TM@9mo (n=13) | 28.12±3.82 | 32.55±4.66 | 0.153±0.064* |
| cKO noTM (n=8) | 27.01±4.54 | 30.21±5.23 | 0.110±0.080** |
| cKO TM@9mo (n=8) | 25.73±1.93 | 26.19±1.79 | 0.016±0.044^a,b^ |

Differences between groups were determined by one-way analysis of variance (ANOVA) followed by Bonferroni post hoc test. *P<0.05 versus CTL noTM, **P<0.01 versus CTL noTM, ^a^P<0.001 versus CTL noTM, and CTL TM@9mo and ^b^P<0.05 versus cKO noTM.
