# Supplementary material for: Elimination of huntingtin in the adult mouse leads to progressive behavioral deficits, bilateral thalamic calcification, and altered brain iron homeostasis
Source: PLoS Genet. 2017 Jul 17;13(7):e1006846. doi: 10.1371/journal.pgen.1006846 (PMC5536499; doi:10.1371/journal.pgen.1006846)
Supplement: S7 Table — Testes from control (noTM and TM-treated at 9 months of age) and of cKO (noTM and TM-treated at 9 months of age) were collected from male mice ranging from 18 to 22 months of age, fixed in 4% PFA and weighed. For each animal examined, both testes were weighed and the mean was determined and used as absolute value for each mouse. Data are expressed as mean ± SD and n = number of mice examined. One-way analysis of variance (ANOVA) followed by Bonferroni post hoc test, ***P<0.001 versus CTL noTM, CTL TM@9mo and cKO noTM. (DOCX) [file pgen.1006846.s019.docx]

**S7 Table. Testicular atrophy in mice lacking Htt.**

| Age | Weight in mg |
| --- | --- |
| 18-22mo CTL noTM | 116.25 ± 11.71 (n=8) |
| 18-22mo CTL TM@9mo | 108.25 ± 1.71 (n=4) |
| 18-22mo cKO noTM | 116.70 ± 6.61 (n=5) |
| 18-22mo cKO TM@9mo | 67.35 ± 7.35*** (n=6) |

Differences between groups were determined by one-way analysis of variance (ANOVA) followed by Bonferroni post hoc test,  ***P<0.001 versus CTL noTM, CTL TM@9mo and cKO noTM.
