# Supplementary material for: Elimination of huntingtin in the adult mouse leads to progressive behavioral deficits, bilateral thalamic calcification, and altered brain iron homeostasis
Source: PLoS Genet. 2017 Jul 17;13(7):e1006846. doi: 10.1371/journal.pgen.1006846 (PMC5536499; doi:10.1371/journal.pgen.1006846)
Supplement: S8 Table — Organs from female and male mice from different cohorts were weighted as described. Data are represented as mean ± SD and n = number of mice examined. Except for brain and testes no significant differences were observed in other organs. (DOCX) [file pgen.1006846.s020.docx]

**S8 Table. Organ weights**

|  | 15-18mo Females TM@6mo | | 15-18mo Males TM@6mo | |
| --- | --- | --- | --- | --- |
|  | CTL (n=6) | cKO (n=6) | CTL (n=6) | cKO (n=6) |
| Body weight (g) | 29.17±4.29 | 22.95±2.79* | 43.03±7.46 | 27.58±3.53** |
| Brain (mg) | 531.38±7.50 | 475.03±11.77*** | 529.35±8.22 | 475.83±14.17*** |
| Heart (mg) | 167.72±18.59 | 158.42±16.31 | 180.62±22.72 | 164.28±35.99 |
| Kidney (mg) | 241.00±30.51 | 236.17±14.27 | 264.83±19.51 | 266.52±30.90 |
| Liver (mg) | 1459.11±260.73 | 1485.42±226.22 | 1736.45±222.07 | 1863.33±413.06 |
| Lungs (mg) | 263.35±53.81 | 275.52±25.10 | 321.95±81.58 | 294.65±135.02 |
| Spleen (mg) | 81.92±15.31 | 72.48±16.48 | 75.78±15.34 | 74.73±21.63 |
| Testes (mg) | N/A | N/A | 116.52±5.68 | 71.98±9.84*** |

Statistically significant differences between pairs (CTL TM@6mo and cKO TM@6mo of each sex group) were determined by Student’s t-test. *P<0.05; **P<0.01; ***P<0.001. N/A: non applicable.
