# Supplementary material for: Elimination of huntingtin in the adult mouse leads to progressive behavioral deficits, bilateral thalamic calcification, and altered brain iron homeostasis
Source: PLoS Genet. 2017 Jul 17;13(7):e1006846. doi: 10.1371/journal.pgen.1006846 (PMC5536499; doi:10.1371/journal.pgen.1006846)
Supplement: S9 Table — Cortices from 18mo CTL TM@6mo and 18mo cKO TM@6mo mice were analyzed as described in Methods. Data are expressed as mean ± SD, and n = number of mice examined. No significant differences were observed. (DOCX) [file pgen.1006846.s021.docx]

**S9 Table. Htt elimination does not alter cortical thickness or calbindin neuronal numbers.**

|  | 18mo CTL TM@6mo | 18mo cKO TM@6mo |
| --- | --- | --- |
| Cortex thickness (μm) | 1,200.4±33.5  (n=6) | 1173.8±23.9  (n=6) |
| Calbindin-positive neurons | 76±2.6  (n=3) | 77±11.2  (n=3) |

Data are expressed as mean ± SD, and   n=number of mice examined. No significant differences were observed.
