# Supplementary material for: Elimination of huntingtin in the adult mouse leads to progressive behavioral deficits, bilateral thalamic calcification, and altered brain iron homeostasis
Source: PLoS Genet. 2017 Jul 17;13(7):e1006846. doi: 10.1371/journal.pgen.1006846 (PMC5536499; doi:10.1371/journal.pgen.1006846)
Supplement: S10 Table — Striata from 18mo CTL TM@6mo and 18mo cKO TM@6mo mice were analyzed as described in Methods. Data are expressed as mean ± SD, and n = number of mice examined. No significant differences were observed. (DOCX) [file pgen.1006846.s022.docx]

**S10 Table. Htt elimination does not alter striatal medium spiny neuronal numbers.**

|  | 18mo CTL TM@6mo  (n=3) | 18mo cKO TM@6mo  (n=3) |
| --- | --- | --- |
| DARPP-32-positive neurons | 98.1±13.4 | 97.0±11.2 |
| Calbindin-positive neurons | 91.7±10.8 | 93.8±14.6 |

Data are expressed as mean ± SD, and   n=number of mice examined. No significant differences were observed.
